# Nanoparticle-Mediated *In Situ* Molecular Reprogramming of Immune Checkpoint Interactions for Cancer Immunotherapy

**DOI:** 10.1021/acsnano.1c04456

**Published:** 2021-10-22

**Authors:** Adam A. Walters, Gemma Santacana-Font, Jin Li, Nadia Routabi, Yue Qin, Nathalie Claes, Sara Bals, Julie Tzu-Wen Wang, Khuloud T. Al-Jamal

**Affiliations:** † Institute of Pharmaceutical Science, Faculty of Life Sciences & Medicine, King’s College London, Franklin-Wilkins Building, 150 Stamford Street, London SE1 9NH, United Kingdom; ‡ EMAT, University of Antwerp, Groenenborgerlaan 171, 2020 Antwerp, Belgium

**Keywords:** SNALP, mRNA, siRNA, immunotherapy, immune checkpoint

## Abstract

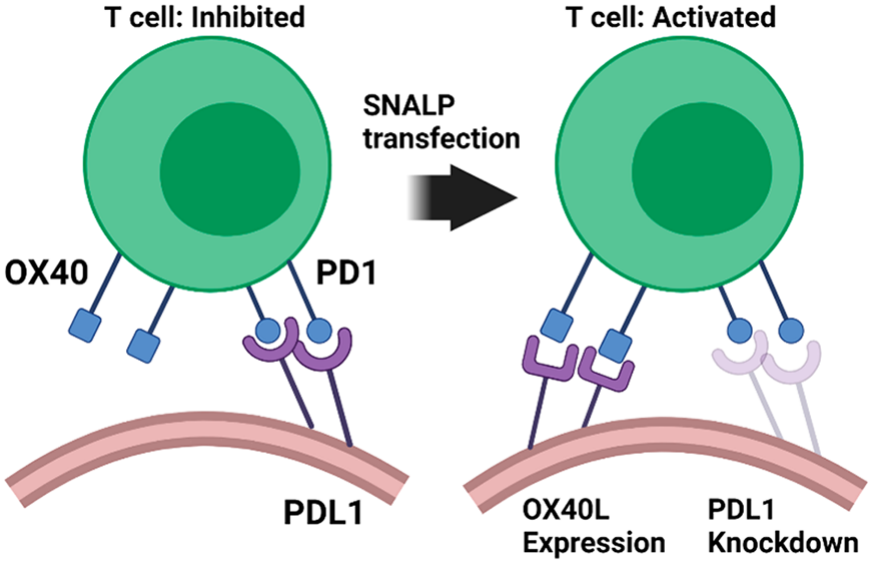

Immune checkpoint blockade involves targeting immune regulatory molecules with antibodies. Preclinically, complex multiantibody regimes of both inhibitory and stimulatory targets are a promising candidate for the next generation of immunotherapy. However, in this setting, the antibody platform may be limited due to excessive toxicity caused by off target effects as a result of systemic administration. RNA can be used as an alternate to antibodies as it can both downregulate immunosuppressive checkpoints (siRNA) or induce expression of immunostimulatory checkpoints (mRNA). In this study, we demonstrate that the combination of both siRNA and mRNA in a single formulation can simultaneously knockdown and induce expression of immune checkpoint targets, thereby reprogramming the tumor microenvironment from immunosuppressive to immunostimulatory phenotype. To achieve this, RNA constructs were synthesized and formulated into stable nucleic acid lipid nanoparticles (SNALPs); the SNALPs produced were 140–150 nm in size with >80% loading efficiency. SNALPs could transfect macrophages and B16F10 cells *in vitro* resulting in 75% knockdown of inhibitory checkpoint (PDL1) expression and simultaneously express high levels of stimulatory checkpoint (OX40L) with minimal toxicity. Intratumoral treatment with the proposed formulation resulted in statistically reduced tumor growth, a greater density of CD4+ and CD8+ infiltrates in the tumor, and immune activation within tumor-draining lymph nodes. These data suggest that a single RNA-based formulation can successfully reprogram multiple immune checkpoint interactions on a cellular level. Such a candidate may be able to replace future immune checkpoint therapeutic regimes composed of both stimulatory- and inhibitory-receptor-targeting antibodies.

## Introduction

Immune checkpoint blockade is a type of immunotherapy based on the use of monoclonal antibodies to block suppressive “checkpoints”; these are regulatory interactions between cells that moderate the immune response.^1^ In cancerous conditions these signals are detrimental as they prevent immune rejection of the tumor. Furthermore, the tumors may actively hijack this axis by overexpressing regulatory molecules on the cell surface to suppress local immune responses. Currently, antibodies raised against PD1/PDL1 and CTLA4 are licensed for use in clinical practice.^2^ PD1 is highly expressed on activated T cells and interacts with its ligands, PDL1 and PDL2, expressed on antigen-presenting cells, inflamed tissue, and some cancer cells; the outcome of this interaction is suppression of T cell activity. Blocking these interactions has been extremely successful, with nine anti-PD1/PDL1 antibodies marketed for 16 cancer conditions and many currently being trialed.^3^ Recently, there has been interest in the development of antibodies targeting co-stimulatory molecules to activate the immune system; such molecules include OX40, 4-1BB, and CD80/86. In contrast to inhibitory checkpoints, the interaction of stimulatory checkpoints serves to promote the immune response though a number of potential mechanisms, such as increased proliferation and activation.^4^ Rationally targeting multiple checkpoints, both stimulatory and inhibitory, with antibodies has been shown to give synergistic effects.^5^ However, the use of antibodies to this end may be limited due to cost, safety concerns, and, importantly, the requirement of colocalization to be most effective.^5,6^

RNA offers an alternative to antibodies as it can both downregulate immunoinhibitory molecules (siRNA) or encode immunostimulatory ligands (mRNA).^7^ An RNA-based approach may be advantageous over antibodies as it is generally cheaper and easier to manufacture. RNA-based approaches have been used to deliver siRNA against multiple targets, including surface molecules such as PDL1 and CTLA4, as well as intracellular molecules, such as IDO and SOCS1.^8−11^ Multiple siRNA constructs have also been coformulated; for example, combinations of siCD47 and siPDL1 in a lipid-based formulation resulted in significant tumor growth reduction compared to either monotreatment.^12^ In parallel to the rise of siRNA, mRNA has been used to express costimulatory molecules including OX40L, CD80, CD86, and numerous cytokines.^13^ For example, the combined use of mRNAs encoding OX40L, IL-23, and IL-36γ resulted in durable immunity in several tumor models.^14^

One of the most exciting prospects of RNA-based immune checkpoint blockade is the potential to “reprogram” checkpoint interactions of individual cells within tumors from an immunosuppressive to immunostimulatory phenotype through the simultaneous delivery of both siRNA (e.g., PDL1) and mRNA (e.g., OX40L). The use of a single formulation to vector both constructs and the necessity of transfection ensures a spatiotemporal relationship is established on a cellular level. Furthermore, the colocalization of immune checkpoint blockade to the tumor through *in situ* delivery can increase potency with reduced off target effects, which have been observed in antibody approaches.^15^ Despite the successful use of combined stimulatory/inhibitory antibodies in various preclinical settings and the existence of both mRNA and siRNA for immune checkpoint blockade, to date there has never been a successful demonstration of a combinatory approach using mRNA/siRNA.^16,17^

To achieve this outcome, as both mRNA and siRNA are unstable in the body and are only active once reaching the cytosol, they must first be formulated with a suitable carrier. Examples include polycations, lipid, or polymeric particles.^18−20^ The stable lipid nanoparticle (SNALP) platform is becoming the preferred means to deliver the RNA and has been used with siRNA in several human clinical trials.^21^ SNALPs are typically composed of ionizable and structural lipids, a PEGylated lipid, and cholesterol.^22^ The ionizable lipid, which is positively charged at low pH, enables association with the negatively charged RNA during formulation while being near neutrally charged at physiological pH ensuring biocompatibility. Following endocytosis and acidification of the endosome, the ionizable lipid becomes protonated and interacts with anionic lipids causing the endosomal membrane to be disrupted and nucleic acid to be released to the cytosol.^23^ This mechanism allows for high transfection efficiency with low toxicity.

This study seeks to validate a dual-targeting approach via concurrent delivery of siRNA/mRNA in a single formulation based on a SNALP platform. We selected to target PDL1 for knockdown, via siRNA, and OX40L for overexpression, via mRNA. In choosing this combination, we speculate that the removal of PDL1-mediated immune suppression will enable the activation and proliferation of T cells receiving stimulation from the T cell receptor and CD80/86. The addition of OX40L co-stimulation will sustain T cell proliferation and enhance survival as has been shown in the literature.^24^ The simultaneous knockdown and expression of PDL1 and OX40L, respectively, will hereby reprogram the tumor toward an immunostimulatory state. When used as a therapeutic intervention, this formulation will increase tumor immunogenicity resulting in delayed tumor growth.

## Results and Discussion

### Validation of *In Situ* Molecular Reprogramming Using Commercially Available Transfection Reagents

Prior to production of our SNALP formulations, we first established whether it was physiologically feasible to both knockdown PDL1 and express OX40L in B16F10. These targets were selected based on their strong representation within the literature as in-depth target validation was beyond the scope of this study. The use of mOX40L has been pioneered by the leading mRNA biotherapeutic manufacturer Moderna Therapeutics.^14^ Their mOX40L construct has been tested alongside other mRNA constructs resulting in potent immune activation. As such, it represents a perfect candidate to validate the *in situ* molecular reprogramming approach.^13,14^ The siPDL1 construct has been used in various forms such as PEI and lipid-based particles, in numerous preclinical models, including B16F10, with promising efficacy.^18,25^

Knockdown and expression was demonstrated to be possible using commercial transfection reagents, plasmid DNA (pOX40L), and siRNA (siPDL1). As shown in Supplementary Figure 1A,B, PDL1 expression could be reduced by up to 50% with siPDL1, and furthermore, OX40L expression could be induced to an MFI of 420 with pOX40L. We also wished to establish whether PDL1 and OX40L would be suitable targets for therapy *in vivo*; to address this, an animal experiment was carried out as outlined in Supplementary Figure 1C. As shown in Supplementary Figure 1D, mice receiving both siPDL1 and pOX40L bore significantly smaller tumors at the end of the study compared to either monotreatment. These differences, while significantly different, were slight and potentially not of therapeutic relevance. Of the monotreatments, pOX40L had the most pronounced effect, and the monotreatment of PDL1 has no/little effect. The published synergy between these two molecules is controversial, with some studies suggesting an enhanced effect and others suggesting no synergy.^26,27^ Other data has suggested that there may be a more subtle, temporal relationship.^28^ In keeping with our data, in a recent study a nanoparticle has been used to codeliver both anti-OX40 and anti-PD1 antibodies. The particulate codelivery resulted in superior immune stimulation when compared to free antibodies, strongly suggesting a spatial relationship is important.^6^ In our study, the beneficial effects of the combined approach were observed despite a relatively small quantity of nucleic acid being used. The nucleic acid dose was limited as precipitation of the complex was observed even at the low doses used. Moreover, PEI is associated with toxicity at higher doses.^29^

### Development of SNALPs Containing Both mRNA and siRNA for *In Situ* Molecular Reprogramming

**Scheme 1:**
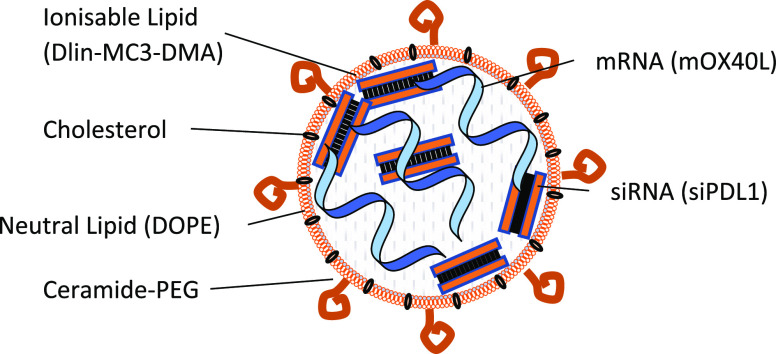
Development of a mRNA/siRNA Dual-Encapsulating SNALP Suitable for *In Situ* Molecular Reprogramming This project describes the development of a SNALP system for co-encapsulation of mRNA and siRNA. The proposed formulation is composed of ionizable lipid, cholesterol, neutral lipid, and PEG ceramide surrounding the relevant nucleic, mRNA and/or siRNA, and acid pay load. A graphical representation is shown.

Having validated potential targets, PEI was substituted for a SNALP-based system. The SNALP system was selected to circumvent toxicity/formulation issues as described for PEI and for translational relevance. The ionizable lipid Dlin-MC3-DMA was chosen due to its availability, published potency, and its clinical application.^30^ Many alternate lipid systems have been proposed including lipidoid-based systems, these may offer improved transfection or loading.^31,32^ The pDNA was substituted for mRNA (mOX40L) due to the clinical acceptability of mRNA and its proximity to translation. SNALPs were prepared using previously published lipids and formulation parameters optimized for mRNA delivery. The proposed scheme for the SNALP structure is shown in Scheme 1. We aimed to produce SNALPs with a size no larger than 200 nm and with maximized encapsulation efficiency (EE%). As shown in Table 1, the size of the SNALPs was only slightly affected by the nucleic acid payload and ranged from 143 to 149 nm. The SNALPs bore a net positive charge (∼16 mV) when the measurement was carried out in citrate buffer (pH 4) diluted with water and this was unaffected by nucleic acid content. The positive charge could be attributed to the fact that at low pH the ionizable lipid is protonated. When buffer was exchanged for PBS (pH 7.4) the charge was near neutral. A high encapsulation efficiency was achieved in all cases: SNALPs incorporating siRNA had the lowest efficiency at ∼81%, whereas the combination SNALP had the highest at ∼93%. We speculate that the formulation parameters, including lipid composition and manufacturing methods, are the major size limiting factor. The use of a microfluidic-based system has produced smaller particle with greater loading and allows for future scale up.^33^

**Table 1:** Physicochemical Characterization of RNA-Loaded SNALPs^a^

type of RNA	size (*d*, nm)^b^	PDI^b^	charge at pH 7.4 (mV)^b^^,^^c^	charge at pH 4 (mV)^b^^,^^d^	EE loading efficiency (%)^e^
siRNA	143.85 ± 2.39	0.22 ± 0.02	+2.39 ± 0.12	+16.02 ± 2.31	81.81 ± 5.57
mRNA	144.54 ± 4.22	0.19 ± 0.02	+0.4 ± 3.09	+16.58 ± 3.08	87.05 ± 9.37
mRNA–siRNA	149.16 ± 3.06	0.22 ± 0.02	–0.45 ± 0.14	+16.53 ± 2.20	93.66 ± 0.59

a *n* = >4 SNALPs per sample.

b Size, polydispersity, and charge were measured with Zetasizer (Malvern Instruments).

c Measured in citrate buffer (pH 4) diluted 1:200 in deionized water.

d Measured in phosphate buffer 0.1 M (pH 7.4)

e The encapsulation efficiency (EE %) was measured with the RiboGreen assay.

**Figure 1:**
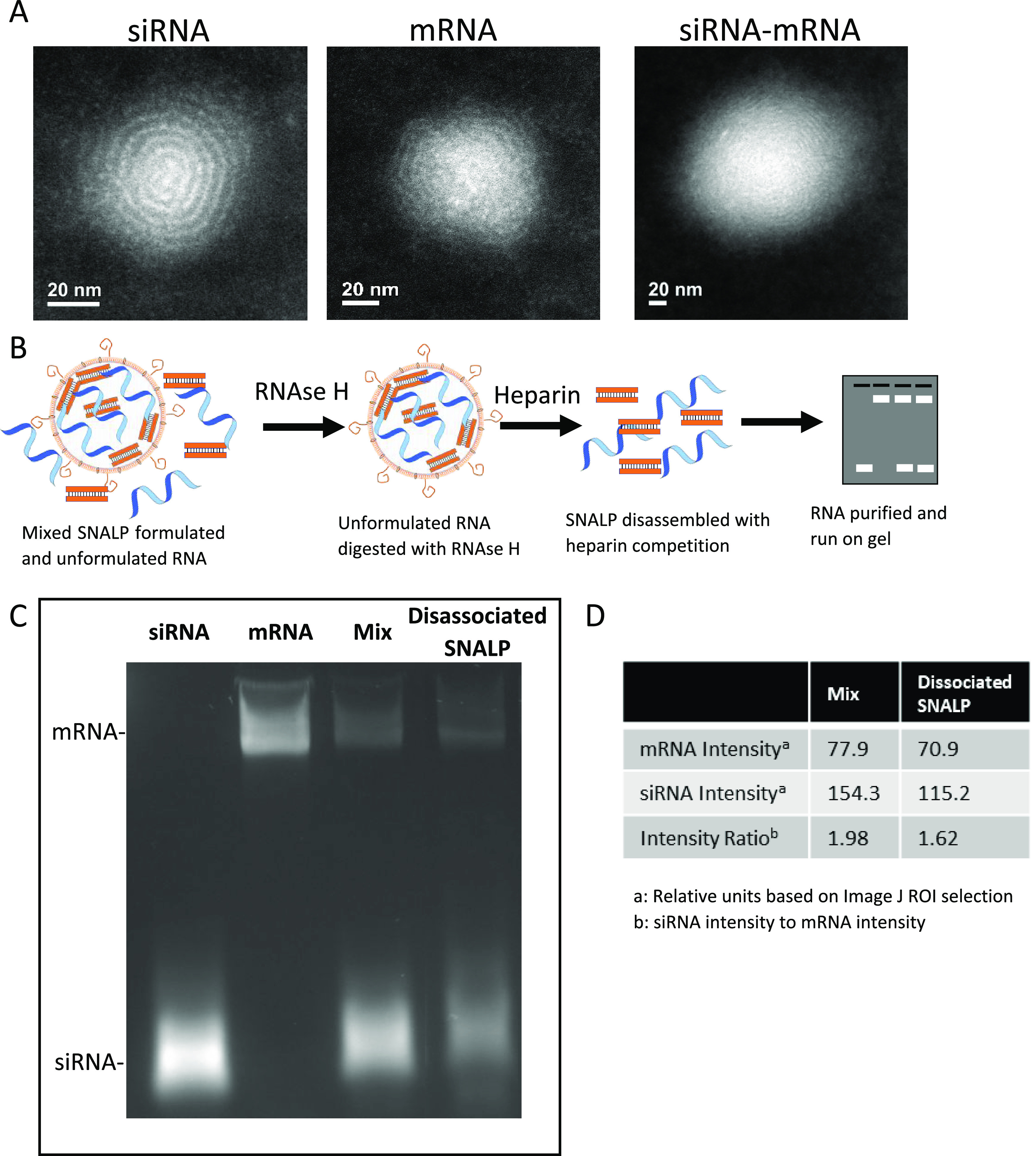
SNALPs have an irregular structure with evidence of internal concentric rings and both mRNA and siRNA are loaded into SNALPs with minimal interference between molecules. (A) SNALPs were formulated with siRNA, mRNA, or a combination of both RNA molecules as previously described. SNALPs were drop-cast on to a graphene grid and imaging was carried out using a Tecnai Osiris transmission electron microscopy. Images represent a single event representative of the wider field. (B) To confirm that both types of nucleic acid can be loaded into SNALPs and that there is minimal hindrance between either molecule, SNALPs coformulating mRNA, and siRNA were treated with RNase H (1 mg/mL) to degrade non-encapsulated/external RNA. The enzyme was inactivated with heat and EDTA (1.25 mM), the SNALP was dissociated by incubation with 10% (*v*/*v*) heparin. The RNA was purified with Monarch RNA Cleanup Kit and run on a 2% agarose gel at 225 V for 25 min. (C) Resulting gel image. Free siRNA and mRNA were run as size markers. A mix of the two free nucleic acids (Mix) corresponding to the starting ratio of nucleic acid (50:50) at a quantity equal to the amount obtained from the SNALP was run alongside the RNA extracted from the SNALP (dissociated SNALP). (D) The intensities of the bands for both Mix and dissociated SNALP were measured using imageJ software.

TEM revealed that SNALPs have an irregular shape with a complex internal structure. Visually, there was no discernible evidence of the different nucleic acids affecting SNALP morphology or formation (Figure 1A and Supplementary Figure 2.). This irregularity may be due to the fact that SNALPs were produced using a simple mixing method rather than the controlled micromixing of a microfluidic system known to produce regular particles.^34^ As RiboGreen assay, used to calculate loading, cannot distinguish mRNA and siRNA, there was a concern that should there be preferential loading of a single type of RNA this would be undetected. To address this, the unloaded RNA in the SNALP preparation was digested with RNase H, SNALPs were disassociated with heparin and the nucleic acid contents run on an agarose gel (Figure 1B). Alongside the SNALP contents, unformulated nucleic acids at a comparable ratio to the preformulation ratio (50:50) were also run, as shown in Figure 1C. Although this is a semiquantitative assay, and cannot be used to accurately quantify RNA, a comparable intensity ratio of RNA bands (siRNA/mRNA) was obtained for the RNA mix (∼1.98) and the dissociated SNALP (∼1.62) (Figure 1D). This suggests that the starting ratio of nucleic acids is maintained following formulation and RiboGreen quantification of total RNA is a suitable method for both mRNA and siRNA for future studies. Additionally, it demonstrates that the RNA content of the SNALP can be protected from nuclease attack.

### SNALPs Transfect B16F10 Cells and Result in Simultaneous Expression of OX40L and Silencing of PDL1 with Minimal Toxicity

**Figure 2:**
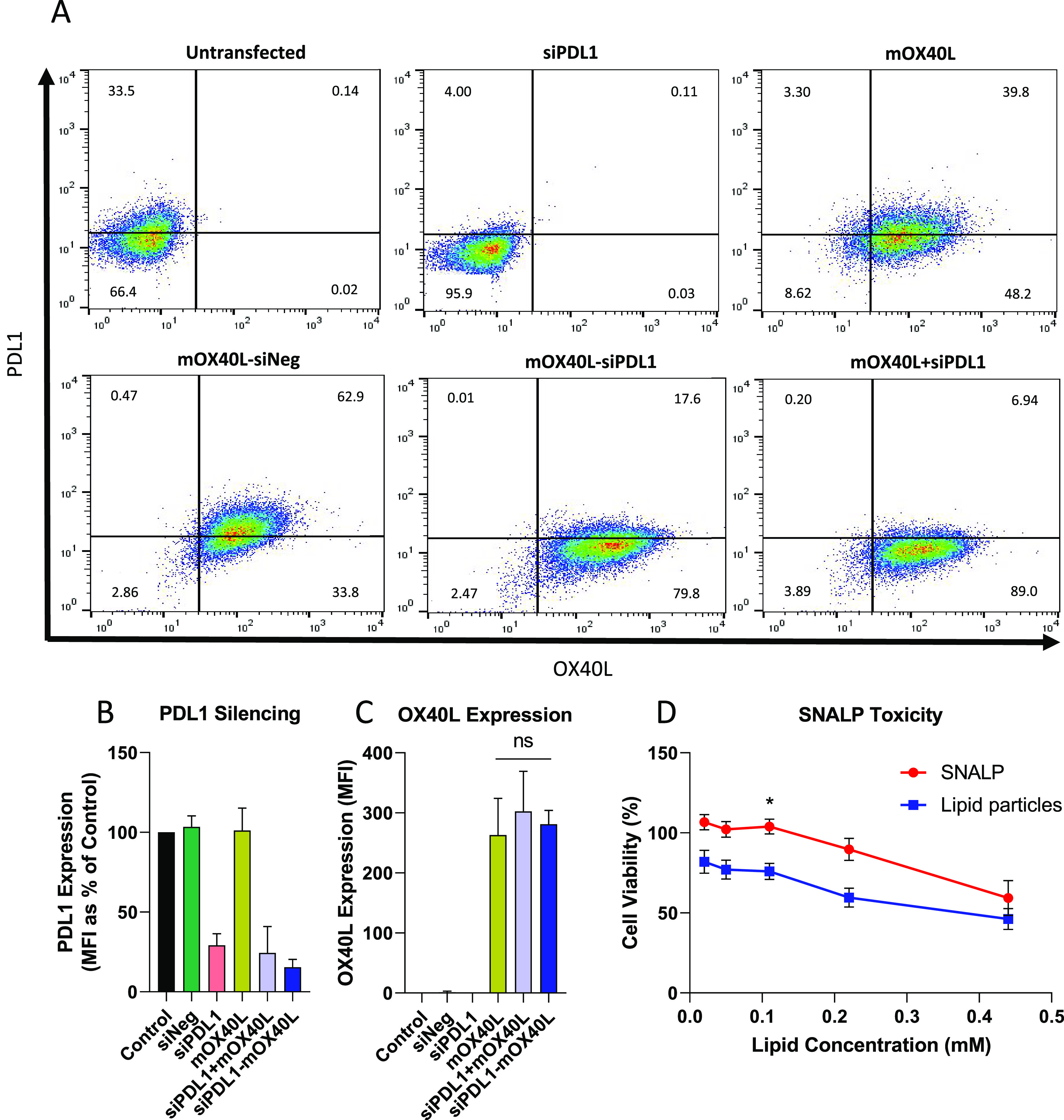
Dual-targeting SNALPs can efficiently transfect B16F10 melanoma cells *in vitro* and display minimal toxicity. B16F10 cells were cultured until 90% confluent before being pulsed with SNALP formulations (0.75 μg of each type of RNA) for 48 h at 37 °C. Cells were harvested and doubly stained with fluorescently labeled anti-mouse OX40L and PDL1 monoclonal antibodies. (A) Shows representative flow cytometry plots. The conditions are as follows: untransfected, siPDL1, mOX40L, mOX40L–siNeg (coformulation), mOX40L–siPDL1 (coformulation), and siPDL1 + mOX40L (mixture of two SNALPs). Quadrant gates were drawn based on isotype control antibody staining, percentage of cells in each quadrant is inset. (B) Shows the values obtained for PDL1 silencing, expressed as MFI percentage of control normalized to 100%. OX40L expression (MFI) is shown in (C). For all the graphs, error bars correspond to standard error of the mean (SEM). Significance was examined with one-way ANOVA multiple comparison test (Tukey’s); *n* = 3–8 repeats for each SNALP formulation. (D) To assess viability of B16F10 cells after being pulsed with SNALPs or RNA-free lipid particles an MTT assay was carried out. A 2-fold dilution series of test formulations was prepared and incubated with cells for 48 h at 37 °C. Error bars were drawn by standard error of the mean (SEM) average of *n* = 10, significance was tested with a two-way ANOVA: Sidak’s multiple comparison test. *,*p* < 0.05.

To determine whether the SNALP formulation was able to transfect cells *in vitro*, B16F10 cells were incubated with SNALPs containing nucleic acids for 48 h. SNALPs containing siNeg, siPDL1, mOX40L, mOX40L–iNeg (coformulation), mOX40L–siPDL1 (coformulation), and siPDL1 + mOX40L (mixture of two SNALPs) were used. Representative flow cytometry plots are shown in Figure 2A; gates were drawn based on isotype controls. As shown in Figure 2A, B16F10 cells express PDL1 at moderate levels and do not express OX40L under normal conditions (untransfected). In all cases, following transfection, cells behave as a single homologous population. For the untransfected cells and for cells treated with SNALPs containing mOX40L, there were 66.40% (lower quadrants) and 56.82% (lower quadrants) of B16F10 viable cells that did not express PDL1, respectively. However, when cells were treated with SNALPs containing siPDL1 alone or in the presence of mRNA, a reduction in the PDL1+ population is observed. For SNALPs entrapping siPDL1, 95.90% of cells were negative for PDL1 (lower quadrant); for coformulated SNALPs and SNALP mixture, 82.27 and 92.89% of the viable cells were negative for PDL1, respectively. This qualitative shift indicates that there has been a silencing in the expression of this marker. Figure 2B shows the downregulation of PDL1 relative to the control in terms of MFI; in all cases, treatment of cells with siPDL1 containing SNALPs resulted in the reduction of PDL1 expression levels to 25% of the control. PDL1 downregulation was comparable whether siRNA was used in isolation (siPDL1) or formulated with mOX40L in two SNALPs (siPDL1 + mOX40L) or coformulated (siPDL1–mOX40L). The PDL1 downregulation was shown to be siRNA-specific, rather than a byproduct of transfection, as siNeg did not induce any downregulation (Supplementary Figure 3).

In terms of mOX40L transfection, as shown in Figure 2A untransfected cells and siPDL1 SNALPs-treated cells did not express OX40L. Upon transfection with SNALPs encapsulating mOX40L alone, coformulated with siNeg, coformulated with siPDL1, or treated with a mixture of SNALPs, 88.00, 96.70, 97.40, and 95.94% of the cells were induced to express OX40L. In terms of MFI, transfection of cells with mOX40L resulted in high expression of the protein and was independent of whether the mRNA was used in isolation or formulated with siPDL1 (Figure 2C). Simultaneous delivery of siRNA and mRNA was previously carried out in a lipid-based system using methods similar to those outlined in this manuscript. In this work it was found that the inclusion of mRNA or alternate polyanions aided the siRNA in silencing.^31^ In our system we observed comparable silencing in the presence or absence of mRNA. We speculate that this discrepancy maybe due to a number of factors. In the previous work, a lipidoid was used in place of an ionizable lipid. It is possible that the lipidoid binds to RNA with a higher affinity than ionizable lipid; thus, the release of siRNA is aided by the presence of a polyanion which reduces the affinity for siRNA by neutralizing some of the charge. Alternately, in our system, the RNA concentration was not titrated, and we may see more obviously an effect of the coloading at lower concentrations. Future studies may comprise of further optimization of nucleic acid ratios similar to work which has been carried out for constructs delivering multiple plasmids.^35^

The viability of B16F10 melanoma cells after SNALPs transfection was assessed with a quantitative MTT assay. For this experiment, a 2-fold dilution series of lipid preparations ranging from 0.02 to 0.44 mM, with or without RNA was tested. As shown in Figure 2D, the viability of B16F10 cells was 100% for those cells that were transfected with SNALPs encapsulating RNA (+RNA) at a concentration of lipid that ranged from 0.02 to 0.1 mM. At 0.22 mM RNA-containing SNALPs, the B16F10 viability was 89.30%, whereas at a concentration of 0.44 mM, viability further decreased to 59.30%. The RNA-free lipid particles (−RNA) were more toxic than the formulated at the lowest dose (82.0% viability compared to 100%). At 0.05, 0.11, 0.22, and 0.44 mM, the viability was 77.10, 75.90, 59.50, and 46.13% respectively. Therefore, from this experiment, it can be concluded that the cell viability decreases as the concentration of the lipid rises and that the SNALPs encapsulating RNA are significantly less toxic than the lipids alone. As a reference, the concentration of SNALPs used during the transfection experiments was between 0.05 and 0.11 mM, depending on nucleic acid content, which is within the nontoxic range. Taken together, these data show that simultaneous upregulation of OX40L and downregulation of PDL1 can be achieved using a single SNALP system with no detectable interference and that SNALPs are relatively nontoxic on a cellular level. To validate the *in vivo* potential of SNALPs, B16F10 cells were also transfected with luciferase expression mRNA (mLuc) in the SNALP system in the presence of serum as previously described. The presence of serum reduced the expression of luciferase by 51% (Supplementary Figure 4); however, cells were still readily transfected resulting in the high expression of luciferase protein.

### SNALPs Can Transfect a Macrophage Cell Line Resulting in Expression of OX40L and Activation

**Figure 3:**
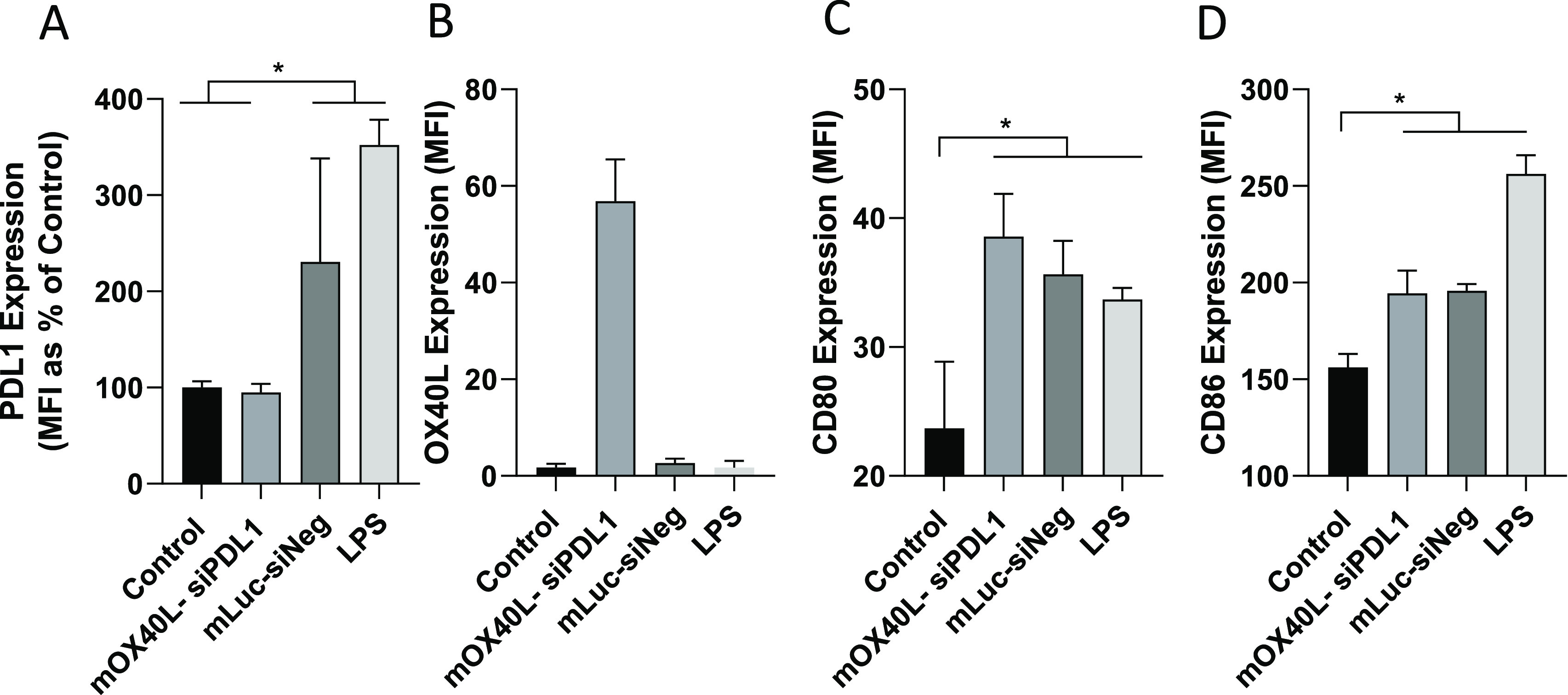
SNALPS can transfect J774 macrophage *in vitro* resulting in activation. To assess effect of SNALPs on macrophage/APC populations J774 cells at 100 000/per well were cultured in a 12-well plate prior to addition of SNALPs loaded with mOX40L–siPDL1 or mLuc–siNeg (1 μg/well) as a negative control. Lipopolysaccharide (LPS) (1 μg/mL) was added as a positive maturation control. Cells were harvested after 48 h at 37 °C and stained with fluorophore labeled anti mouse PDL1 (A) and OX40L (B) monoclonal antibodies or anti-mouse CD80 (C) or CD86 (D). Cells were acquired on FACs Calibur flow cytometer. To analyze staining, cells were first gated by FSC/SSC profile before the relevant marker was assessed. Error bars correspond to the SD statistical analysis was carried out using Mann–Whitney test. *, *p* < 0.05.

Macrophages comprise a large proportion of tumor-associated cells, representing up to 50% tumor weight.^36^ Furthermore, they are highly phagocytic, thus representing an additional target for transfection following *in situ* administration. To model the effect of transfection on phagocytes/APCs, a J774 mouse macrophage cell line was selected. The J774 macrophage line was incubated with SNALPs containing either siPDL1–mOX40L or siNeg–mLuc. LPS was included as a positive control for macrophage activation. As shown in Figure 3A, treatment of J774 with siNeg–mLuc containing SNALPs or LPS resulted in a 2- or 4-fold upregulation of PDL1 respectively. Treatment of cells with siPDL1–mOX40L containing SNALPs negates this upregulation, and PDL1 levels remain comparable to the untreated control. Consistent with the results obtained for B16F10 cells, OX40L expression could be induced only by SNALPs containing siPDL1–mOX40L, though the relative MFI was lower than that obtained for B16F10 cells (Figure 3B). To test whether SNALPs can upregulate the expression of maturation markers, the relative expression levels of CD80 and CD86 were tested (Figure 3C,D). Cells receiving either SNALP formulation or LPS had a CD80 expression 1.75-fold higher than the untreated control and were not significantly different from each other. Likewise, CD86 was also upregulated by both SNALPs to a similar extent, though LPS was more potent in this regard. Combined, this suggests that siPDL1–mOX40L SNALPs may be able to activate macrophages while also inhibiting the upregulation of PDL1 and inducing expression of OX40L.

### Intratumoral Administration of SNALPs Leads to Transfection within the Tumor

**Figure 4:**
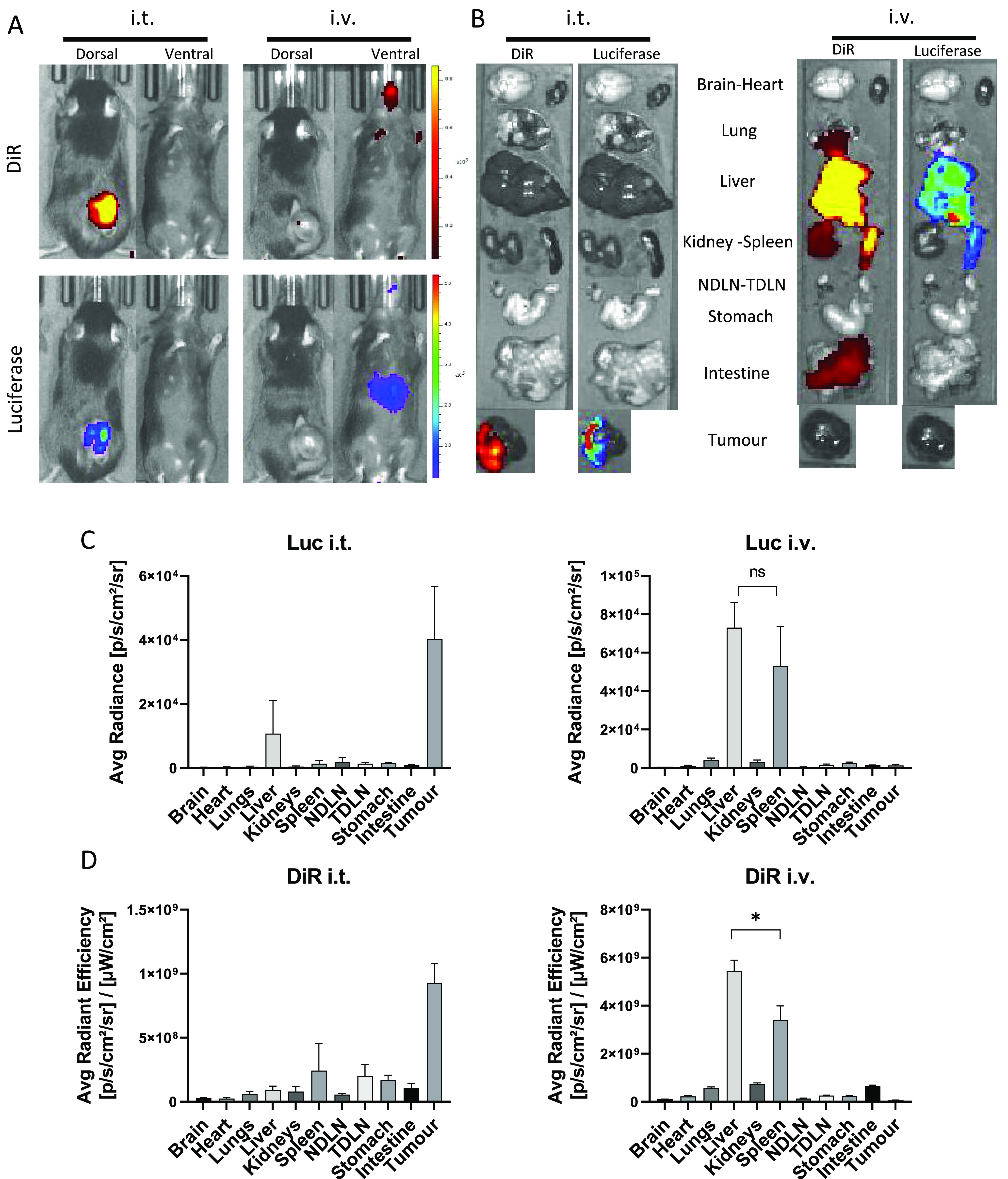
Biodistribution and *in vivo* expression of mLuc following administration of RNA-loaded, DiR-labeled SNALPs via two different routes. C57BL/6 (*n* = 4 per group) were implanted with 1 × 10^6^ B16F10. On day 13 postimplantation, mice were injected with SNALPs formulated with 1% DiR and containing 13 μg of mLuc per mouse either i.v. or i.t. in 100 or 50 μL volume, respectively. One mouse was left untreated to serve as a negative control. (A) At 4 h postinjection, whole body imaging was carried out to assess both luminescence and DiR fluorescence (ex.745 nm, em. 800 nm) using an IVIS Spectrum *in vivo* imaging system. (B) Following imaging, mice were sacrificed, and organs were extracted and imaged as described above. For each of the images a single representative is shown. Organ images were analyzed using Living image software; DOI were drawn around each organ manually and both average radiance for luciferase expression (C) and average radiant efficiency for DiR (D) were plotted. In each case, the mean ± SD of the group is shown. Statistical analysis was carried out using a Mann–Whitney test. ns, nonsignificant; *, *p* < 0.05.

Following the positive *in vitro* transfection, the biodistribution and *in vivo* transfection was assessed. To perform this, SNALPs were formulated containing mLuc and a lipid intercalating dye (DiR) to measure transfection and distribution, respectively. As siRNA and mRNA are both active in the cytosol and as the *in vitro* data shows they do not hinder the activity of each other, mRNA was used in isolation as a measure of transfection. Two injection routes were tested, the more clinically acceptable intravenous (i.v.) route, following which the particles would reach the tumor through the enhanced permeation and retention effect (EPR), and the direct intratumoral (i.t.) route, which had given positive results in the preliminary study. Mice were imaged at 4 h postinjection based on previous reports on mRNA *in vivo* transfection.^37^ Representative images following whole body imaging are shown in Figure 4A. Administration of SNALPs i.t. led to strong DiR and luciferase signal from the tumor area, with the two signals corresponding in both location and relative intensity. Bioluminescence signals could be detected from the liver region in the i.v. group when the mice were imaged ventrally but not dorsally. In contrast, there is minimal DiR signal detected in the same group. This is due to the inability of fluorescence to penetrate the animal from deeper tissues. Results obtained from *ex vivo* analysis of the individual organs were in general agreement with the whole body imaging results (Figure 4B). Using the i.t. injection route, both luciferase and DiR signals were detected in the tumor. Luciferase expression within the tumor followed the DiR distribution pattern. As expected, i.v. administration resulted in a more disseminated biodistribution with organs such as the intestine and lungs giving positive DiR signals over background. Whether this was due to organ deposition of SNALPs, or SNALPs remaining in the vasculature, it could not be determined. It should be noted that no luciferase transfection of these organs was detected. The organs giving the strongest DiR and luciferase signals following i.v. injection were the liver and the spleen. The signals obtained for luciferase and DiR were then normalized per organ and are shown in Figure 4C,D. Quantitative transfection data (Figure 4C), in agreement with *ex vivo* organ imaging, showed that luciferase signals were highest in the tumor of mice after i.t. injection of SNALPs, while no signals were detected after i.v. administration. The i.v. administration resulted in the highest signals in the liver and the spleen (*p* > 0.05). Organ biodistribution quantitative data (Figure 4D) further confirmed that SNALPs were retained in the tumor following i.t. injection and in liver/spleen following i.v. injection (liver > spleen, *p* < 0.05). We did not observe tumor targeting following i.v. administration which had been expected due to the EPR effect.^38^ This could have perhaps been foreseen as SNALPs of a similar composition have been used to deliver RNA to the liver, mediated by Apo E targeting.^39,40^ There are recent studies showing that the modification of the lipid components of the nanoparticle can result in selective organ targeting, for example, the inclusion of permanently charged cationic lipids (DOTAP) results in strong splenic targeting; this approach has not been tested for tumors but may make for an interesting future study.^41^ The shape of the particles has likewise been shown to impact tumor uptake following i.v. administration, with star-shaped particles showing a higher uptake in tumors.^42^ However, what we understand of the EPR effect and passive tumor targeting is undergoing a radical shift with a growing body of evidence suggesting that a particulate nature alone is unsuitable for tumor targeting in the clinic.^43^ SNALP accumulation in the spleen, a secondary lymphoid tissue, following i.v. injection could potentially be used to enhance systemic immune responses. Improved tumor targeting may be observed following i.v. injection if a targeting moiety is added as has been described.^44^ Due to this observation, we opted to continue with the local, intratumoral approach. Intratumoral therapy has been gaining prominence recently, with a number of high impact, preclinical studies having demonstrated efficacy.^14,45^ Moreover, there are several clinical trials either underway or having been completed assessing the suitability of this route as a clinical option for delivering mRNA or pDNA.^46−49^ It is based on the premise that the tumor itself can serve as a “vaccine” (i.e., a source of antigen) should the i.t. immune stimulation be potent enough (so-called *in situ* vaccination).^50^ While this is an unconventional route, should it prove efficacious and technically realistic, it represents a promising approach allowing for delivery of concentrated immunotherapeutics to the tumor site potentially increasing potency and circumventing systemic toxicity.

### Both Immune and Nonimmune Cells Contribute to Uptake of SNALPs following i.t. Administration

**Figure 5:**
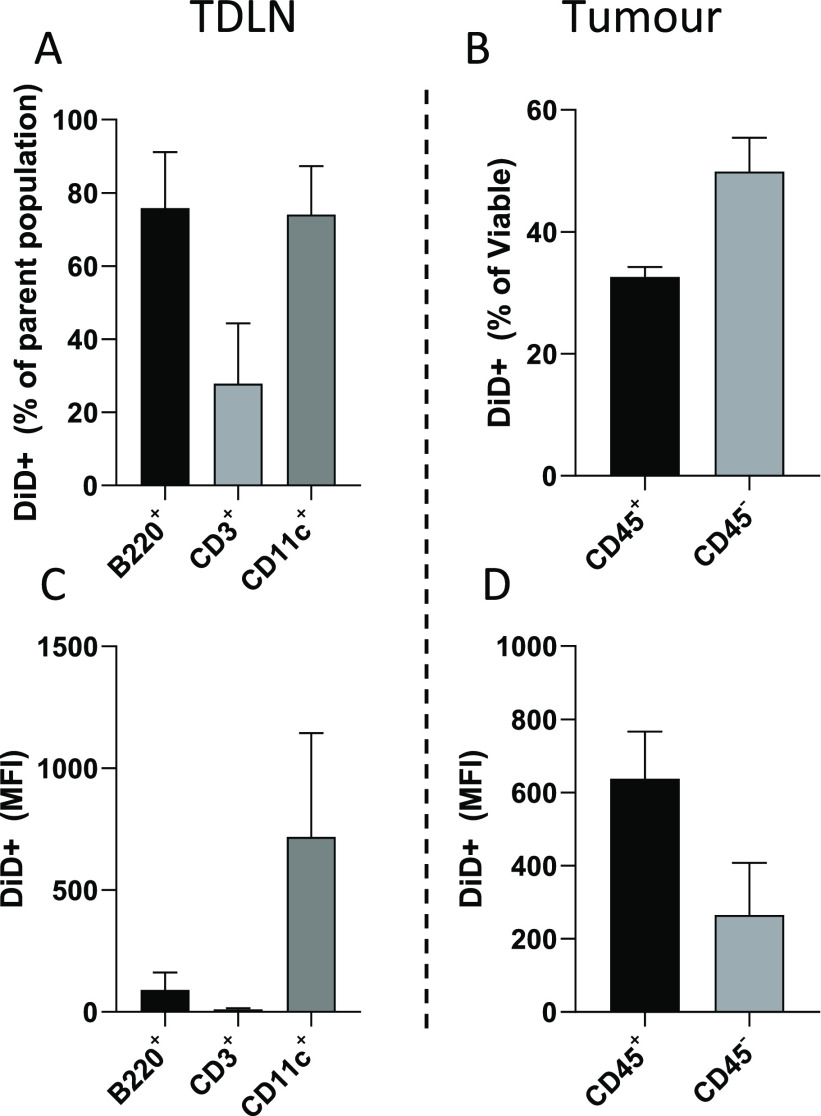
SNALPs are distributed to both immune and nonimmune cells in the tumor and TDLN following i.t. injection. C57/Bl6 (*n* = 4) were implanted with B16F10 cells subcutaneously. Once palpable, tumors formed SNALPs formulated with 1% DiD and containing 13 μg of siNeg were injected i.t., one mouse was left uninjected to serve as a control. At 24 h postinjection mice were culled, and TDLN and tumors were extracted. A single-cell suspension from each of the tissues was obtained by physical maceration. (A, C) Lymph node cells were stained with antimouse B220, CD3, or CD11c. In each case, the percentage of cells of the parent population positive for DiD SNALPs is shown in (A), and the DiD MFI of the whole cell population is shown in (C). (B, D) Cells obtained from tumors were stained with anti-mouse CD45 and PI. The CD45 positive and negative population positive for DiD SNALPs as a percentage of viable is shown in (B) the DiD MFI of the whole cell population is shown in (D). Error bars correspond to SD.

To test cellular distribution of SNALPs within the tumor and TDLN, mice bearing B16F10 tumors were i.t. injected with fluorescently labeled (DiD) SNALPs containing nonspecific RNA. After 24 h, cells obtained from tumors and TDLN were examined by flow cytometry. Interestingly, despite being undetectable using IVIS whole body or *ex vivo* organ imaging (data not shown), a substantial amount of SNALPs signal was detected in the TDLN with 74–75% of B220+ (B cells) and CD11c+ (APCs) cells showing positive association (Figure 5A,C). Only 27% of the CD3+ cell (T cells) population showed association with SNALPs. In this study, the MFI signal represents the number of SNALPs associated with a cell population on a per cell basis. As shown in Figure 5C, the CD11c+ cells had the highest MFI (mean 714), approximately 8-fold higher than B220+ cells (mean 90), suggesting that these cells had the highest affinity for SNALP association.

Among the viable cells extracted from the tumor, ∼32% of the CD45+ (leukocytes) population was associated with SNALPs, and ∼49% of the SNALPs was associated with other cell populations (CD45- including B16F10 cells) (Figure 5B). The intensities of the SNALPs uptake, expressed as the MFI, were 637 and 265, for the CD45+ and the CD45– populations, respectively (Figure 5D). The data combined suggest that in terms of cell proportions CD45– cells were responsible for the majority of the cellular SNALP association. However, they became associated with the SNALPs to a lesser extent than CD45+ cells. It could be speculated that this discrepancy maybe due to the relative abundance of cells, with CD45– cells outnumbering CD45+ cells.

Following i.t. administration, SNALPs were associated with both immune and nonimmune cells (CD45+ leukocytes vs CD45– cells), though it should be noted we did not establish whether transfection was achieved equally in both. There is an ongoing debate as to whether the tumor itself is the target of immune checkpoint blockade or whether it is the “host” immune cells.^51,52^ The observation of SNALP uptake in CD45+ cells in the tumor microenvironment may indicate that SNALPs can also be used to target tumor-associated immune cells. Indeed, we have shown *in vitro* that SNALPs can transfect a macrophage cell line. To capitalize on this, SNALPs may also be developed to target tumor-associated macrophages delivering mRNA/siRNA to switch them from the nonprotective M2 to the protective M1 phenotype.^53^

### *In Situ* Molecular Immune Checkpoint Reprogramming Results in Significantly Reduced Tumor Growth and the Establishment of Immunostimulatory Conditions

**Figure 6:**
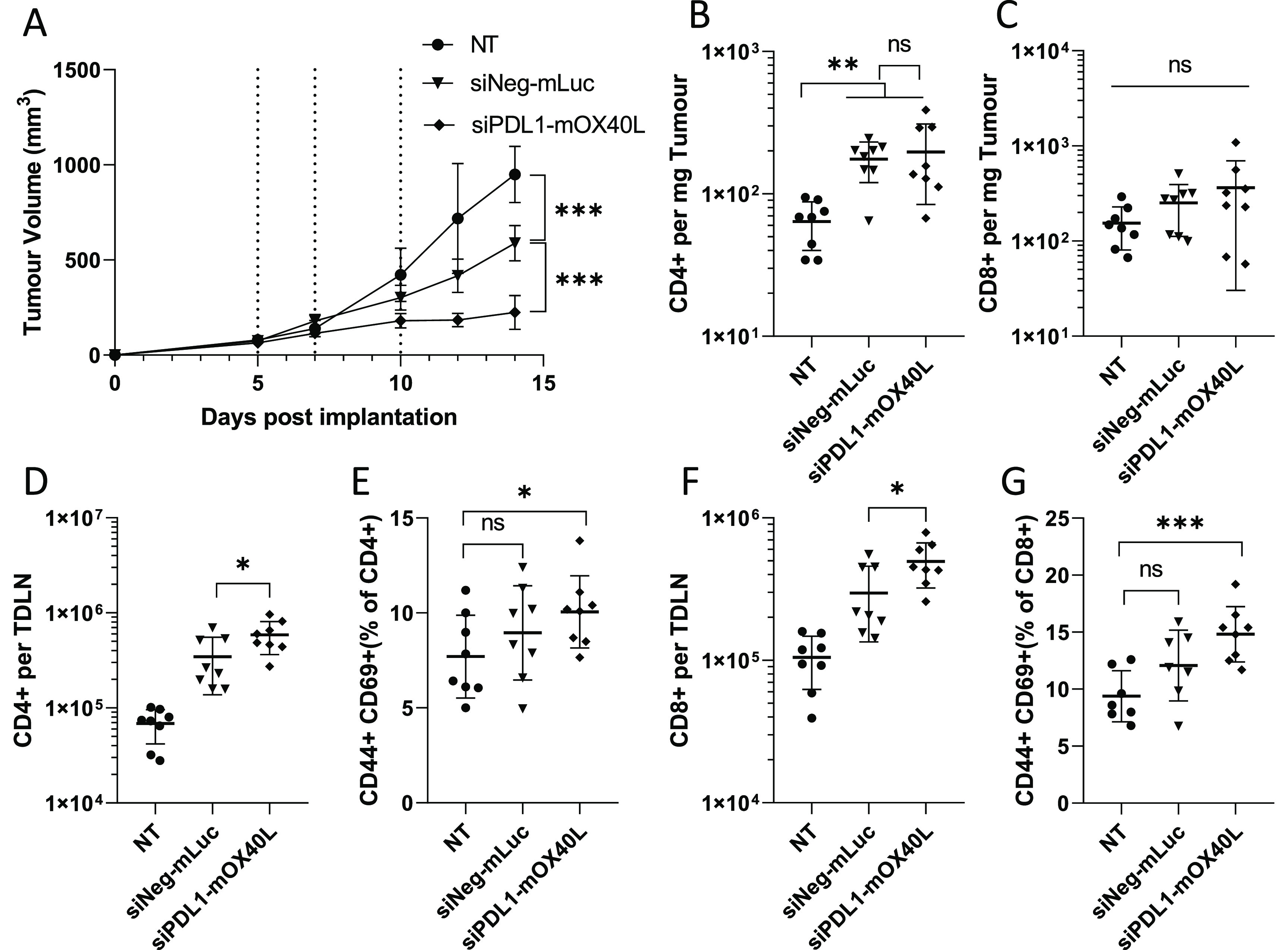
SNALPs containing mOX40L and siPDL1 significantly delay tumor growth and alter leukocyte populations in both tumor an TDLN. C57/Bl6 (*n* = 8 per group) were subcutaneously implanted with B16F10 cells (1 × 10^6^ cells/mouse). Once tumors were palpable (day 5 post implantation), SNALPs containing either mOX40L and siPDL1 or mLuc and siNeg were injected intratumorally (13 μg of total RNA per dose) or left untreated (NT). The SNALPs were administered two further times (days 7 and 10). (A) Tumor growth was monitored over the time course, injection time point is indicated by dotted lines. The group mean ± SD is shown in each case. Once the control group reached its humane end point mice were culled, and tumors and TDLNs were isolated. Single-cell suspensions were obtained using physical dissociation of tissues. Cells extracted from the tumors were stained with monoclonal antibodies targeting CD4 (B) and CD8 (C). Cells obtained from the TDLN were stained with anti-CD4, -CD44, and -CD69 (D, E) or anti-CD8, -CD44, and -CD69 (F, G). Absolute cell counts were obtained by including precision counting beads prior to acquisition on flow cytometer. For tumors, the cell count is normalized to tumor weight (B, C); for TDLN, it is presented as the whole cell fraction obtained from the TDLN (D, F). Lymphocyte activation in the TDLN was assessed by first gating on either CD4 or CD8 before the CD44+, CD69+ dual-positive population was identified. Data are presented as CD44+ CD69+ as percentage of the parent population. Each point represents an individual mouse; error bars correspond to the SD. Statistical analysis was carried out using a Student’s *t*-test. *, *p* < 0.05; **, *p* < 0.005; ***, *p* < 0.001; ns, nonsignificant.

The therapeutic potential of mRNA/siRNA SNALP was investigated in the B16F10 tumor model. A three-dose regime was employed, in keeping with previous preliminary studies. SNALPs containing mLuc and siNeg were used as a negative control (negative SNALP). As shown in Figure 6A, a significant reduction in tumor growth, compared to negative SNALP and to the untreated group, was observed in the group receiving dual siPDL1–mOX40L-targeting SNALP. At the final time point, tumors in the dual-targeting SNALP group were approximately 50 and 80% smaller than the negative SNALP and the untreated group, respectively. We observed unpredicted therapeutic efficacy in the negative SNALP group, which had significantly smaller tumors than the nontreated control at the final time point. To attempt to dissect the immune response to the SNALPs, we analyzed cell infiltrates in the tumor and alterations of lymph node populations.

In Figure 6B,C, a significantly elevated CD4+ cell numbers were observed in tumors of groups receiving SNALPs compared to those in nontreated control. While this general trend was maintained in the CD8+ cell populations, it did not achieve statistical significance due to variation in the data. When TDLN were analyzed (Figure 6D,F), significantly higher numbers of CD4+ and CD8+ cells were observed in the group receiving siPDL1–mOX40L SNALPs compared to the negative SNALPs and the nontreated group with the following order observed: nontreated < negative SNALP< siPDL1–mOX40L. The activation of cells was also assessed using CD44 and CD69 expression. CD44 is an indicator of antigen experience and is commonly used as a memory marker, while CD69 is an early activation marker. Combined, they may indicate ongoing/recent antigen-specific activation. As shown in Figure 6E,G, the general trend, in terms of CD44- and CD69-positive cells as a percentage of parent population (CD4+ or CD8+), is comparable to the trend observed in total cell numbers. However, statistical significance could only be achieved when comparing siPDL1–mOX40L SNALP group with the nontreated group. The negative SNALP group was not statistically different from the nontreated group.

The efficacy observed in our therapeutic model was initially surprising as the B16F10 model is generally considered to be immunologically barren.^14^ We speculate that this is potentially due to two factors: the *in situ* reprogramming of immune checkpoint interactions and the relatively high dose of nucleic acids we used in this study. Using flow cytometry, a number of observations were made of the therapeutic group when compared to the untreated: higher densities of CD4+/CD8+ cells within the tumor, increased levels of CD4+/CD8+ cells in the TDLN, and a greater degree of activation. Combined, these data strongly suggest reprogramming of the tumor to an immunostimulatory phenotype with the dual-targeting SNALP was responsible for the reduction of tumor growth. Potentially, the most unexpected result of the study was the relative therapeutic efficacy of the mLuc–siNeg SNALP. Though this construct was not as potent as the mOX40L–siPDL1, there was a significant inhibition of tumor growth when compared to the untreated tumors. We speculate this may be due to the RNA serving as an immune adjuvant. The observed upregulation of maturation markers in J774 cells *in vitro* may be used as evidence to support this claim. It is possible to further speculate that the inclusion of two types of RNA, single-stranded mRNA and double-stranded siRNA, can activate multiple nucleic acid sensors in immune cells including TLRs 3 and 7/8, MDA-5, and RIG-I.^54,55^ The engagement of multiple nucleic acid sensors in some models has been shown to induce synergistic responses when compared to individual receptors.^56^ The distribution of the SNALPs to the lymph node following i.t. injection would have further enhanced the adjuvant effect as B cells and DCs possess an abundance of the aforementioned nucleic acid sensors. In some previous studies, mRNA was synthesized with the use of pseudobases to reduce activation by the RNA backbone which may explain the discrepancy between our data and published works.^13,57^ An alternate hypothesis may be that the luciferase molecule itself is acting as a foreign antigen which, when combined with the immunostimulatory nucleic acid, may result in immune activation or that there is some adjuvanticity of the lipid construct.^58^

**Figure 7:**
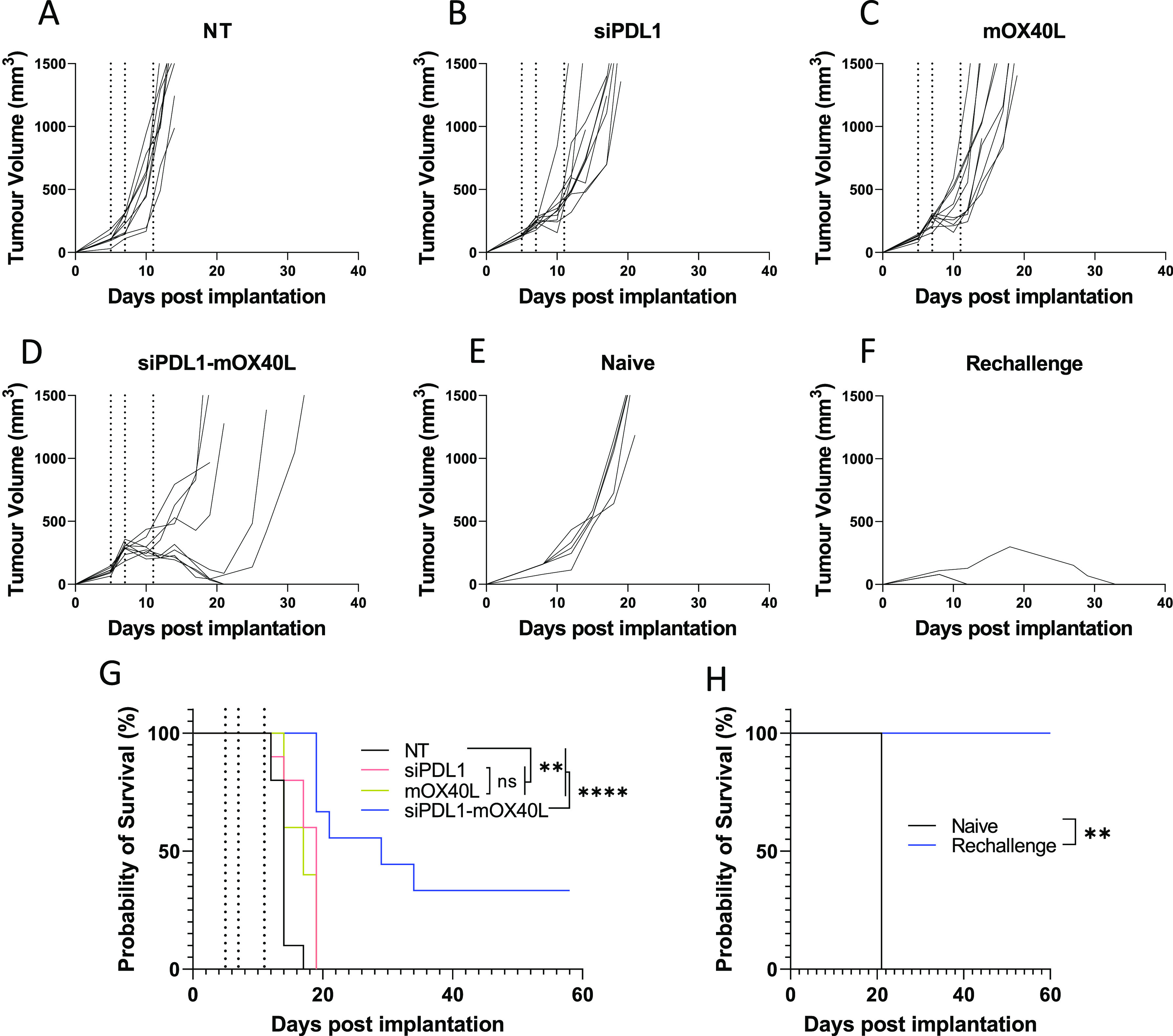
Combinatory SNALPs significantly improves survival compared to mono formulated SNALPs and can afford lasting immunity in a subset of mice. C57/Bl6 (*n* = 9–10 per group) were implanted with B16F10 cells (1 × 10^6^ cells/mouse) subcutaneously. At days 5,7 and 11 post implantation (indicated by dotted lines) tumors were treated i.t. with SNALPs containing either: siPDL1, mOX40L, both mOX40L and siPDL1, or left untreated (NT). Tumor growth was monitored until mice reached their humane end points. The data are presented as a spaghetti plot for individual mice in each treatment groups (A–D). The survival of the mice over the time course is shown as a Kaplan–Meier plot (G). Surviving mice from (G) (*n* = 3) were rechallenged with B16F10 cells contralaterally at 60 days after first implantation; as a control, naïve age-matched mice (*n* = 5) were likewise implanted with B16F10 cells. Tumor growth was monitored, and the growth curves for individual mice in each mouse group is shown in (E) naïve and (F) rechallenge. The survival of the mice is shown in Kaplan–Meier plot (H). Survival curves were analyzed using a Mantel–Cox test. **, *p* < 0.05 ****, *p* < 0.0001; ns, nonsignificant.

Having established the therapeutic efficacy, we next sought to test the combinatory formulation alongside the monoformulations in a long-term survival model. As shown in Figure 7, using the regime previously established we were able to observe tumor growth delays when treated with either monoformulations or combinatory formulations. This was reflected in mouse survival times, both monotreatments resulted in a median survival of 19 and 17 days for siPDL1 and mOX40L groups, respectively. This was significantly different from the 14-day median survival of the control group. Treatment with the combinatory formulation, however, resulted in a median survival of 29 days which was significantly different from both control and monotreatment groups. Furthermore, within the siPDL1–mOX40L group, 30% of mice showed total remission with no tumors being detectable at 60 days postimplantation compared to no remission in any of the other groups. To determine whether the combination treatment generated immunological memory, the surviving mice were rechallenged with B16F10 cells implanted into the contralateral flank. A group of age-matched “naïve” mice was included as a control. As shown in Figure 7, 100% of naive control mice reached their humane end point by day 21, whereas all rechallenged mice survived until day 60, at which point the study was terminated (Figure 7H). The tumor growth was slower in aged mice compared to the younger mice used in the initial study (control group humane end point reached at 21 vs 14 days); this is consistent with our previous observations (unpublished data). The rechallenged mice, 66% (2/3) had minor detectable tumor growth observed which was completely resolved by day 12; the remaining mouse developed a slow growing mass which had resolved by 33 days postimplantation (Figure 7F). Combined these data suggest that the combinatory approach can induce persistent immunological memory. This may be further improved with an optimized dosing regimen.

CT26 is known to be extremely difficult to transfect using ionizable lipids, believed to be due, in part, to a defective endolysosomal system and thus represents a robust challenge to the developed system.^59,60^ As shown in Supplementary Figure 5, we observe no significant reduction in tumor size or improved mouse survival. This suggests that efficacy may be limited to the highly transfectable cell lines such as B16F10. Indeed, the inability of the formulation to improve the median survival of CT26 bearing mice represents a limitation of the system and highlights that the heterogeneity of tumors represents a challenge to intratumoral transfection-based systems. It is likely that within the clinical setting tumors will be unique to the patient and display idiosyncratic transfection capacities. Therefore, future work should comprise of the development of a “universal” formulation able to transfect a diverse range of cancer cell lines. Moreover, preclinical candidates should be tested in range of cell lines to test clinical suitability.

## Conclusion

**Scheme 2:**
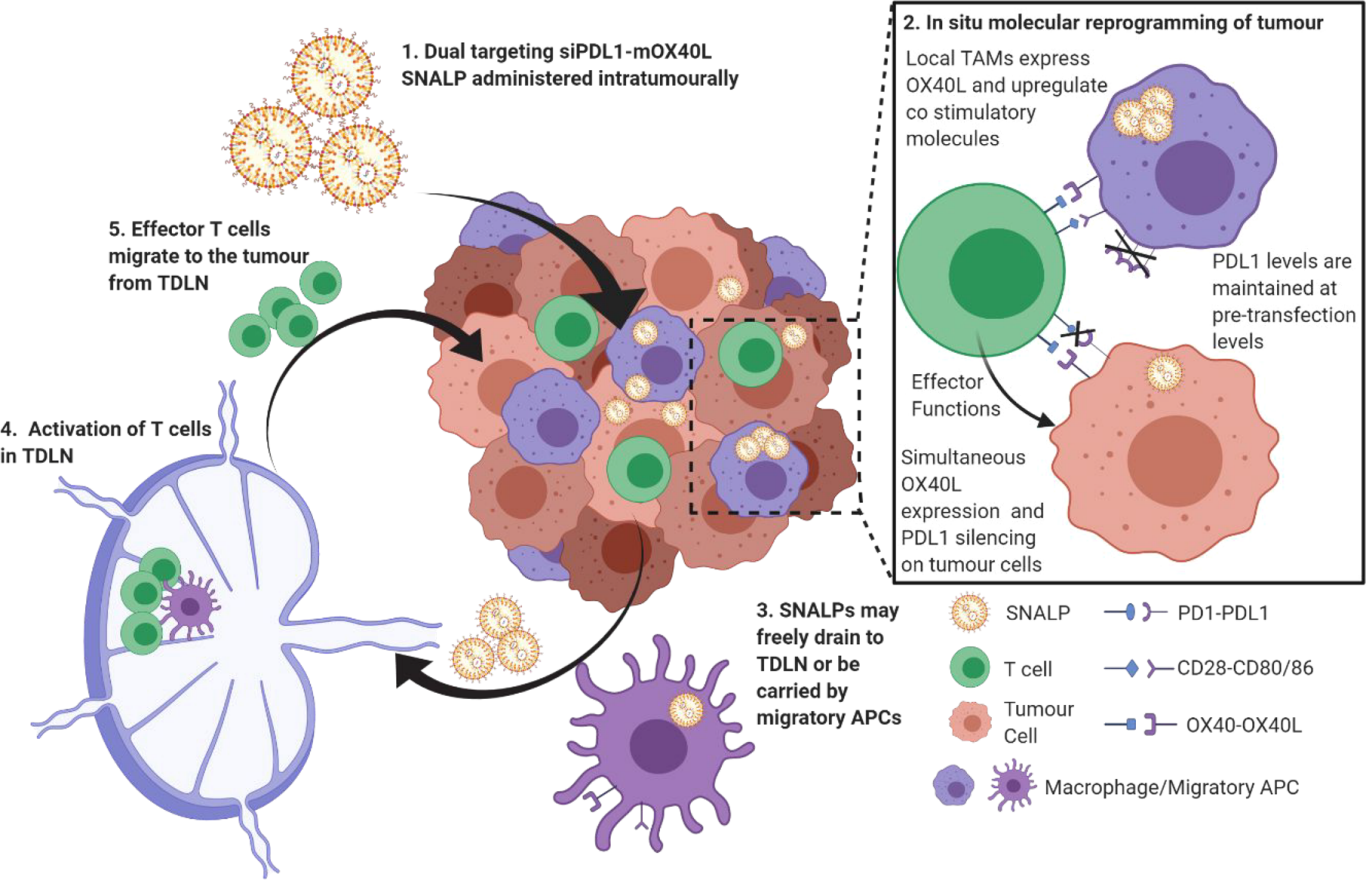
Molecular Programming of Checkpoint Interactions Under diseased conditions, the tumor presents an immunosuppressive microenvironment through, though not exclusively by, expression of PDL1 which limits T cell activation and proliferation. T cell activation by local APCs is likewise limited. Combined, this leads to tumor persistence. Using the formulation proposed within this study, the PDL1-mediated immunosuppression will be removed using siRNA. The tumor will also be induced to express the positive checkpoint molecule OX40L using mRNA. SNALPs will activate APC leading to the expression of a co-stimulatory molecule in addition to OX40L. This results in reprogrammed tumor microenvironment favoring the immunostimulatory state. SNALP may also freely drain to the TDLN of be carried by migratory APC populations resulting in activation and proliferation.

This study outlines an approach to reprogram immune checkpoint interactions by delivering nucleic acids targeting both stimulatory and inhibitory immune checkpoint blockade simultaneously. We have demonstrated that this approach is viable in both *in vitro* and *in vivo* models and our hypothetical mechanism as illustrated in Scheme 2. On the basis of the data obtained, we have evidence to suggest the formulation described herein may be able to replace multi antibody cocktails in future immunotherapeutic regimes.

## Materials and Methods

### Materials

PDL1 siGENOME Mouse CD274 (siPDL1) siRNA-SMART pool was purchased from Dharmacon. The siRNA sequences are GAUAUUUGCUGGCAUUAUA; GAGGUAAUCUGGACAAACA; GAGCCUCGCUGCCAAAGGA; and GAAUCACGCUGAAAGUCAA. A single siRNA sequence, GAGGUAAUCUGGACAAACA, established to be the most potent, was used for animal studies. Nonspecific siRNA with the sequence UGCGCUACGAUCGACGAUG was used as a negative control (siNeg: Eurogentec). Messenger RNA encoding OX40L (mOX40L) was synthesized from a mouse TNFSF4 ORF mammalian expression plasmid (pOX40L). The corresponding noncoding plasmid was used as a negative control (pNeg) (SinoBiological Inc.). Luciferase-pcDNA3 plasmid was a gift from William Kaelin (Addgene plasmid no. 18964), and luciferin was from Promega. For the SNALPs formulation, 1,2-dioleoyl-*sn*-glycero-3-phosphoethanolamine (DOPE) (Lipoid), cholesterol (Sigma-Aldrich), Dlin-MC3-DMA (Bioybt), *N-*palmitoyl-sphingosine-1-succinyl [methoxy (polyethylene glycol) 2000] (C16 PEG 2000 Ceramide) (Avanati), and citrate buffer (pH 4; Sigma-Aldrich) were used. Branched polyethylenimine (PEI) (jetPRIME) was purchased from Polyplus. mRNA synthesis reagents, XbaI, HiScribe T7 ARCA mRNA Kit (with tailing), and Monarch RNA Clean up Kit were purchased from New England Biolabs. Sodium borate was from Santa Cruz Biotechnology; EDTA was from Formedium. Quant-iT RiboGreen, Millennium RNA Markers, and UltraPure Agarose were purchased from Thermo Fisher Scientific. GelRed and lipophilic dyes, 1,1′-dioctadecyl-3,3,3′,3′-tetramethylindodicarbocyanine (DiD) and 1,1′-dioctadecyl-3,3,3′,3′-tetramethylindotricarbocyanine iodide (DiR), were purchased from Biotium. Tissue culture reagents newborn calf serum (FBS), trypsin EDTA, GlutaMAX, RPMI 1640 media, phosphate-buffered saline (PBS), and penicillin–streptomycin were from Gibco, Thermo Fischer Scientific. Methylthiazolyldiphenyl-tetrazolium bromide (MTT) was from Sigma. All additional chemical reagents were purchased from Sigma-Aldrich. All anti-mouse fluorophore-conjugated antibodies (CD8-PE (53–6.7), CD4-FITC/PE (GK1.5), PDL1-PE (10F.9G2), OX40L (RM134L), CD69-APC (H1.2F3), CD3-PE (17A2), CD45-FITC (30-F11), B220-FITC (RA3–6B2), CD44-FITC (IM7), CD11c-APC (N418), CD80-PE (16–10A1), and CD86-PE (A17199A)) were purchased from Biolegend, as was propidium iodide (PI) and True-Nuclear transcription factor staining kit.

### Synthesis of mRNA

OX40L mRNA (mOX40L) was synthesized from a mouse TNFSF4 ORF mammalian expression OX40L plasmid. Luciferase mRNA (mLuc) was synthesized from Luciferase-pcDNA3 plasmid. The plasmid was first linearized with XbaI restriction enzyme, and then the mRNA was synthesized according to HiScribe T7 ARCA mRNA Kit (with tailing) protocol. Finally, mRNA was purified by spin-column with Monarch RNA Clean up Kit. The concentration of mRNA was measured by NanoDrop One (Thermo Fisher Scientific). RNA was assessed by agarose electrophoresis gel on a 1% agarose gel. Agarose and 1× (10 mM) sodium borate (SB) buffer were microwaved until the agarose was completely dissolved, and the solution became clear. Formaldehyde (37%) was added to a final concentration of 0.62%. The gel was transferred into running tank filled with 1× SB buffer, and the Millennium RNA Markers ladder plus the RNA sample was mixed with Gel red and formamide before being loaded into the gel. Finally, the gel was run at 225 V for 40 min and was visualized by illumination under ultraviolet light (GelDoc, Bio-Rad).

### Preparation of SNALPs

To make the SNALPs for each condition, an ethanolic lipid (Supplementary Table 1) and aqueous (Supplementary Table 2) phases were prepared in separate tubes. After prewarming them at 60 °C for 3 min, 10 μL of the lipid phase was introduced into the aqueous phase. The liquid solution was rapidly pipetted up and down for 20 s, vortexed for 10 s, and incubated at 60 °C for 30 s. The process was repeated until all the lipid phase had been added. They were incubated for 1 h at 40 °C and flushed with dry N_2_ to remove residual ethanol. For *in vivo* studies, SNALP buffer was exchanged using Amicon Ultra centrifugal filters (0.5 mL) 30 kDa (Merck Millipore) in accordance with manufacturer’s protocol. SNALPs were resuspended in 200 μL of 4-(2-hydroxyethyl) piperazine-1-ethanesulfonic acid (HEPES) buffer (pH ∼7). The final mass ratio of ionizable lipid to RNA was 10:1 *w*/*w*. To produce fluorescently labeled SNALPs, DiD or DiR at a final molar percentage of 1% was included in the lipid phase. SNALPs were loaded with either nonspecific siRNA (siNeg), siRNA specific to PDL1 (siPDL1), luciferase mRNA (mLuc), or OX40L mRNA (mOX40L) and are named accordingly. When multiple RNAs are combined in a single SNALP, the formulation is named with a dash between the two RNA constructs (e.g., siPDL1–mOX40L). In transfection experiments where multiple SNALPs with differing constructs are utilized, a plus sign is added between the two RNA construct names (e.g., siPDL1 + mOX40L).

### Physico-Chemical Characterization of SNALPs

SNALPs encapsulating different types of RNA were characterized in terms of size (*z*-average), polydispersity, and surface charge (*z*-potential) utilizing dynamic light scattering (DLS). In brief, SNALPs in citrate buffer were diluted with deionized water (1:10 *v*/*v*) and added to a disposable plain folded capillary zeta cell. Measurements were obtained at 25 °C in triplicate using Zetasizer Nano ZS (Malvern Instruments).

### Annular Dark-Field Scanning Transmission Electron Microscopy (ADF-STEM) Imaging

SNALP formulation (5 μL) was drop-cast on to a graphene TEM grid. Annular dark-field scanning transmission electron microscopy (ADF-STEM) was carried out on a Tecnai Osiris TEM operated at 200 kV. STEM images were acquired with 50 pA beam current.

### Determination of Total RNA Encapsulation Efficiency (EE%)

The encapsulation efficiency (EE%) was indirectly quantified with the Quant-iT RiboGreen assay according to manufacturers’ protocol and as described previously.^61^ Ribogreen is an RNA-detection agent, and due to its membrane impermeability when added to a SNALP formulation, it stains only external, nonencapsulated RNA. When a permeabilization agent, such as Triton X-100, is included, Ribogreen can stain interior as well as exterior RNA (total RNA). SNALPs were incubated with PBS or PBS + 0.4% Triton X-100 for 20 min. RNA standards comprising serial dilutions of siRNA in either PBS or PBS + Triton X-100 were prepared as measurement references. Following incubation, RiboGreen was added to each sample, and the fluorescence was measured at ex./em. 485/520 nm with a plate reader (BMG LABTECH, FLUOstar Omega). RNA concentration was interpolated from the relevant standard curve of siRNA diluted in either PBS or PBS plus Triton X-100 accordingly. The quantity of loaded RNA was determined by subtracting the values obtained in PBS (external RNA) from PBS + Triton X-100 (total RNA) as shown below in (eq 1). Encapsulation efficiency was then established based on the amount of RNA encapsulated relative to the preformulation quantity (eq 2). It should be noted that this method detects all RNA and cannot differentiate mRNA from siRNA.
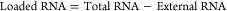
1
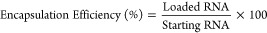
2

### Semiquantitative Assessment of RNA Loading Proportions

Since RiboGreen assay does not discriminate mRNA from siRNA, agarose gel electrophoresis was used to establish mRNA/siRNA loading proportions. A SNALP preparation was incubated with RNase H (20 U/mL) to degrade the unloaded RNA, after 20 min incubation at 37 °C, the enzyme was inactivated by heat (20 min at 65 °C) and by the addition of EDTA to a final concentration of 1.25 mM. The SNALPs were then treated with heparin (10% *v*/*v*) to dissociate the particle; liberated RNA was purified with Monarch RNA Cleanup Kit as per manufacturer’s instructions. Finally, RNA was run on an 1% agarose gel at 225 V for 40 min as described above (see the “Synthesis of mRNA” section). Gel images were analyzed using ImageJ, and manual regions of interest (ROI) were drawn around each band. Band intensities calculated by ImageJ were used to calculate siRNA to mRNA ratio both in the initial mix and the dissociated SNALP. Comparable ratio values would suggest siRNA and mRNA have comparable affinities to SNALP.

### Cell Culture

Mouse melanoma B16F10, CT26, and macrophage J774 cells were maintained in an incubator (Sanyo, MCO-17AIC) at 37 °C, with 5% CO_2_ and with a relative humidity of 5%. The cells were cultured in RPMI-1640 medium supplemented with 10% FBS, 1% penicillin–streptomycin, and 1% GlutaMAX. Cells were treated with 0.05% trypsin–EDTA when they achieved 90% confluency and were passaged every 2–3 days.

### *In Vitro* SNALP Transfection of B16F10 Cell Lines

B16F10 cells were seeded in a 12-well plate at a density of 120 000/well with 1 mL of fully supplemented RPMI-1640 medium 24 h before the transfection and incubated at 37 °C and 5% CO_2_. To perform SNALP transfection, a volume of SNALPs corresponding to 0.75 μg of RNA was diluted with 1 mL of serum-free RPMI-1640. The SNALP containing media (1 mL) was added a confluent well of B16F10 cells in a 12-well plate, after 4 h, the wells were supplemented with FCS to a final concentration of 10% *v*/*v* and incubated for a further 48 h at 37 °C and 5% CO_2_. Transfected cells were stained with anti-PDL1-PE, anti-OX40L-APC, or with their respective isotype antibodies for 20 min at 4 °C before being washed 3 times with PBS and finally resuspended in PBS. Cells were acquired using a FACS Calibur flow cytometry (BD Biosciences). Relative OX40L expression is plotted as the obtained MFI. PDL1 expression was expressed as a percentage Mean Fluorescence Intensity (MFI) values from untransfected control.

### SNALP Transfection of J774 Macrophages

To assess J774 transfection and activation, cells were maintained and cultured as described for B16F10. A volume of SNALPs corresponding to 1 μg was diluted in 1 mL of serum-free tissue culture media and added to a single well of confluent cells. After 4 h of incubation, FCS was added to a final concentration of 10% *v*/*v*; after 48 h, cells were harvested. J774 cells were transfected with SNALPs containing either mOX40L–siPDL1 or mLuc–siNeg as a negative control. An additional group received lipopolysaccharide (LPS) to a final concentration of 1 μg/mL as a stimulation control. Cells were harvested and stained with anti-PDL1-PE and anti-OX40L-APC, as well as anti-CD80-PE and anti-CD86-PE as markers of activation. Data were analyzed using FlowJo software (Treestar); cells were regated based on their FSC/SSC profile before the marker of interest was assessed. Marker expression was presented using their respective MFI values.

### *In Vitro* Cytotoxicity Using MTT Assay

To assess the cytotoxicity of SNALPs, a 96-well plate was seeded with B16F10 (6000 cells/well), and cells were incubated at 37 °C and 5% CO_2_ for 24 h until 90% confluency was achieved. SNALPs encapsulating RNA were added at a range of dilutions starting at 0.44–0.02 mM and incubated in complete media at 37 °C and 5% CO_2_ for 48 h. In parallel, cells were also incubated with lipids without nucleic acid to assess the toxicity of the transfection reagent. Then, the 96-well plate was incubated for 4 h with 120 μL/well MTT working solution (5 mg/mL of MTT solution diluted 1:5 with tissue culture media). Subsequently, DMSO was added into each well, and the plate was incubated for 5 min at 37 °C. Finally, absorbance at 570 nm was measured with the plate reader, and cell viability was calculated by calculating absorbance as a percentage of the untreated cells.

### Mice

Animal experiments were carried out in female C57BL/6 or BALB/c mice (6–8 weeks old, Envigo). All the experiments involving animals were previously approved by the local ethical committee and with the approval of the United Kingdom Home Office license and in accordance with the UKCCCR Guidelines (1998).

### SNALP Biodistribution and *In Vivo* Transfection

To assess organ biodistribution and *in vivo* expression of mRNA, naïve female C57BL/6 mice (*n* = 4 per group) were bilaterally implanted with 10^6^ B16F10 cells. On day 13 postimplantation, mice were injected intravenously (i.v.) or intratumorally (i.t.) with DiR-labeled SNALPs loaded with mLuc (13 μg per mouse) in either 100 or 50 μL of HEPES buffered saline, respectively. One mouse was left untreated as a background control. At 4 h after SNALP injection, mice were injected subcutaneously with luciferin before whole body luminescence and fluorescence (ex. 745 nm; em. 800 nm) imaging on an IVIS Spectrum *in vivo* imaging system (PerkinElmer). Following whole body imaging, mice were sacrificed, and individual organs (brain, heart, lungs, stomach, liver, kidneys, and intestine) were imaged for both luminescence and fluorescence as described for whole body imaging. Data were analyzed using Living Image software (PerkinElmer). Fluorescence and bioluminescence values for individual organs were obtained by manually drawing regions of interest (ROI) around each organ prior to analysis. Data are expressed as the average radiance obtained per ROI for luciferase signals and average radiance efficiency per ROI for fluorescence signals.

### Cellular Distribution of SNALPs in Solid Tumors and Tumor-Draining Lymph Nodes

To further assess the cellular distribution of SNALPs, naive female C57BL/6 mice (*n* = 4) were implanted with 10^6^ B16F10 cells. Once palpable, tumors were i.t. injected with DiD-labeled SNALPs (*n* = 3) corresponding to 13 μg of RNA per dose per mouse. At 24 h postinjection, tumors and tumor-draining lymph nodes (TDLN, inguinal on tumor bearing flank) were excised, and a single-cell suspension was prepared by physically macerating tumors in PBS through a 70 μm cell strainer. Tumor cells were stained with antimouse CD45 (leukocyte marker) and PI as viability dye. Cells obtained from the TDLN were stained with fluorescently labeled anti-mouse CD11c, B220, and CD3 to identify DCs, B cells, and T cells, respectively. Finally, cells were analyzed by flow cytometry as previously described. Data are presented as both percentage of cells showing positive association with SNALPs and relative MFI of the respective populations.

### *In Vivo* Immunomodulatory Activity of SNALP Constructs and Survival Studies

C57BL/6 mice (*n* = 8 per group) were implanted with B16F10 cells as previously described. Once tumors are palpable, SNALPs containing 13 μg total RNA, produced as described, were i.t. injected at days 5, 7, and 11 after tumor implantation. Tumor size and mouse weight were monitored until the terminal humane end point was reached (tumor diameter 15 mm) at which point mice were euthanized. Tumors and TDLN were extracted and processed to obtain a single-cell suspension. Tumor cells were stained with anti-mouse CD4 and CD8 antibodies. TDLN cells were stained with anti-mouse CD4, CD8, CD44 (an antigen memory marker), and CD69 (an early activation marker) antibodies to identify CD4+/CD8+ cells and their activation status. A fixed quantity of precision count beads was added to establish absolute cell numbers obtained. Cells were acquired on a FACs Calibur Flow Cytometer, and data analysis was carried out using FlowJo. For tumor cells, data are presented as total CD4/CD8+ cells number per mg of tumor. TDLN cell data is presented as CD4/CD8+ cell number per TDLN and CD44+/ CD69+ cells as a percentage of CD4 or CD8+ cell population.

To monitor the impact of the SNALP treatment on long-term mouse survival, C57/BL6 mice (*n* = 9–10 per group) were implanted with B10F10 cells as described above. Mice were injected i.t. with formulations containing either: 6.5 μg of siPDL1, 6.5 μg of mOX40L or the combinatory SNALP (6.5 μg of siPDL1 + 6.5 μg of mOX40L). Tumors were measured every other day, and mice were culled at their humane end point (tumor length ≥ 15 mm, weight loss ≥ 10% of pretreatment body weight, or visible signs of distress). Mice which cleared the tumor were rechallenged with B16F10 cells as described for the first implantation, a control group of aged-matched mice which had not been exposed to B16F10 (naïve) was also included. Tumor growth was monitored until the humane end point was reached. As an alternate model, CT26 colon carcinoma cells (1 × 10^6^ per mouse) were implanted into the lateral flank of BALB/c (*n* = 7–8 per group). Once tumors had reached ca. 5 mm in diameter, mice were injected i.t. with siPDL1–mOX40L SNALPs containing 13 μg of RNA or buffer. The injections were repeated, and tumor growth was monitored as described for B16F10 model.

### Data and Statistical Analysis

Numerical data was analyzed using GraphPad Prism 8. Data were first analyzed for normality with a Shapiro–Wilks test, dependent on outcome; data were subsequently analyzed with a Student’s *t*-test with/without Mann–Whitney post-test. Where more than two conditions are being compared, data were analyzed with an ANOVA followed by relevant post-test. A survival curve analysis was carried out using a Mantel–Cox test. The statistical test utilized is indicated in each figure caption. Flow cytometry data was analyzed using FlowJo (version 10, Treestar).

## Supporting Information Available

Methods and supplementary figures: validation of selected constructs; further electron micrographs; specificity of knockdown; transfection in serum; survival of mice bearing CT26 tumors following treatment (PDF)
