# Supplementary material for: Nanoparticle-Mediated In Situ Molecular Reprogramming of Immune Checkpoint Interactions for Cancer Immunotherapy
Source: ACS Nano. 2021 Oct 22;15(11):17549–64. doi: 10.1021/acsnano.1c04456 (PMC8613910; doi:10.1021/acsnano.1c04456)
Supplement: Supplementary file 1 — nn1c04456_si_001.pdf [file nn1c04456_si_001.pdf]

Supplementary Information

Nanoparticle Mediated *in Situ* Molecular Reprogramming of Immune Checkpoint Interactions for Cancer Immunotherapy

Adam A Walters[1,*], Gemma Santacana-Font[1], Jin Li[1], Nadia Routabi[1], Yue Qin[1], Nathalie Claes[2], Sara Bals[2], Julie Tzu-Wen Wang[1] and Khuloud T. Al-Jamal[1*]

1. Institute of Pharmaceutical Science, Faculty of Life Sciences & Medicine, King's College London, Franklin-Wilkins Building, 150 Stamford Street, London SE1 9NH, United Kingdom
2. EMAT, University of Antwerp, Groenenborgerlaan 171, 2020 Antwerp, Belgium.

[*]Corresponding authors. Email: Khuloud.al-jamal@kcl.ac.uk: adam.walters@kcl.ac.uk

Supplementary Methods

In vitro transfection of B16F10 using PEI transfection reagent

B16F10 cells were seeded into a 12-well plate at a density of 120 K/well in fully supplemented RPMI-1640 medium 24 h before the transfection and incubated at 37°C, 5% CO_2. PEI transfection was performed according to manufacturer's protocol. The PEI reagent (3 µL/condition) was mixed with siRNA (1.18 µg/ condition) and/or pDNA (0.8 µg/ condition) in the jetPRIME buffer (100 µL/condition). The mixture was incubated for 15 min at room temperature to form PEI/siRNA-pDNA complexes before being added to cells in 1 mL of complete media and incubated for 48h at 37°C, 5% CO_2.

In vivo target validation using PEI complexes

B16F10 cells 10^6 were implanted subcutaneously into the lower flank of C57BL/6 mice in 100 µL PBS. PEI/ siRNA-pDNA complexes were prepared by mixing 1.6 µg plasmid (pOX40L or pNeg) and/or 1.18 µg siRNA (siPDL1 or siNeg) with 3 µg PEI and jetPRIME buffer, diluted to a total volume of 50 µL, and incubated for 15 mins. On day 5 post implantation, once palpable, tumours were i.t. injected with 50 µL complex formulation per tumour. Additional doses were administered at days 7 and 11 post implantation. Tumour size and mouse weight were monitored until mice reached their humane end points.

In vitro SNALP mLuc transfection of B16F10

SNALPs containing mLuc were prepared as previously described (see: Preparation of SNALPs). B16F10 cells were cultured and transfected as described in the relevant methods section (see: *In vitro* SNALP transfection of B16F10 cell lines). To establish the effect of serum during transfection, cells were transfected in the presence or absence of 10% v/v FCS for 4 h. An additional, untransfected group, was included to normalize the background signal. After elapsed time, conditions lacking serum were supplemented with FCS to final concentration 10% v/v; conditions with serum received complete media in equivalent volume. Following 48h incubation, luciferase assay was performed as described in manufacturers' data sheet. In brief: culture media was removed, and cells were gently washed with PBS, 200 µL of 1x reporter lysis buffer was added to each well before being freeze thawed 3 times. Obtained lysate was clarified by centrifugation (14000 rpm, 5 mins). The quantity of luciferase was detected by adding 100 µL of luciferase to 20 µL of cell lysate and being read immediately using a plate

reader (BMG LABTECH, FLUOstar® Omega). Data is presented as relative light units (RLU) for each condition with the background signal obtained from untransfected wells subtracted.

Supplementary Data

Supplementary Table 1: Lipid phase composition per SNALP preparation

Reagent	Concentration	Volume per prep
DOPE	2 mg/mL	13.25 µL
Cholesterol	2 mg/mL	20 µL
Dlin-MC3-DMA	2 mg/mL	25 µL
C16 PEG 2000	4 mg/mL	3.5 µL
Citrate buffer (pH~4)	20 mM	6.86 µL

Supplementary Table 2: Aqueous phase composition per SNALP preparation

Reagent	Stock concentration	Volume per prep	Volume citrate buffer per prep
siNeg[a]	1.3 mg/mL	3.8 µL	96.2 µL
mRNA [a]	1 mg/mL	5 µL	95 µL

a: when dual mRNA:siRNA SNALP was made nucleic acid volumes of each type of RNA were halved

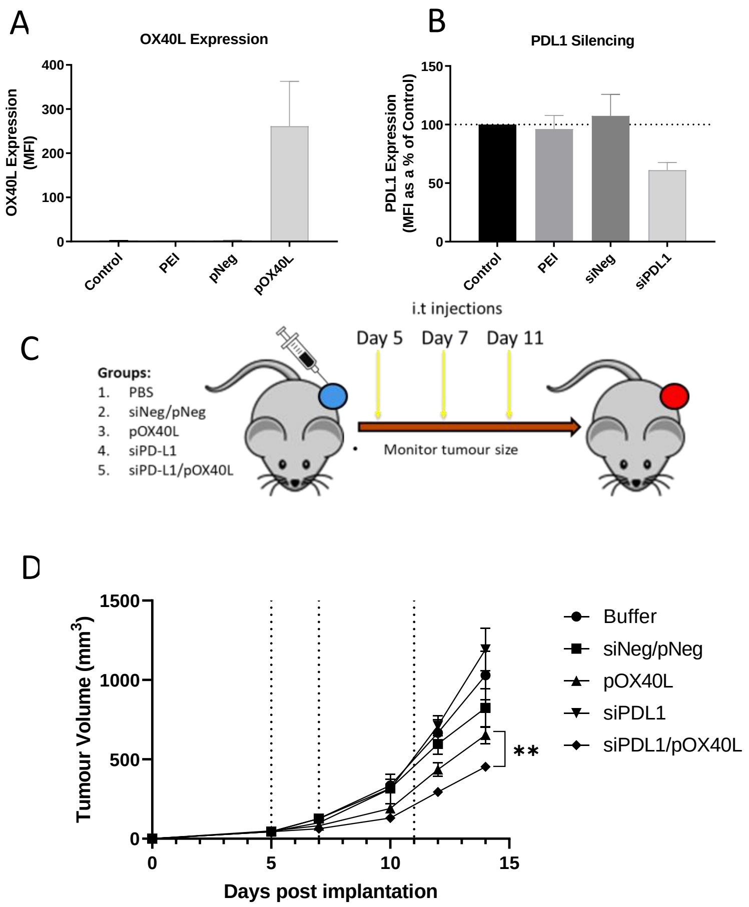

Supplementary Figure 1. Validation of OX40L expression and downregulation of PDL1 using commercial transfection reagents *in vitro* and local, *in vivo*, administration significantly delays B16F10 tumour growth. B16F10 cells were cultured until 90% confluent before being (**A**) transfected with either empty plasmid (pNeg) or plasmid containing OX40L ORF (pOX40L) or (**B**) with either non-specific siRNA (siNeg) or siRNA specific for PD-L1 (siPDL1) in PEI. Cells were left untransfected (control) as negative controls. In all conditions siRNA was fixed at 88nM and pDNA fixed at 0.8 µg. Following 48 hours cells were harvested and stained with fluorescently labelled anti mouse PDL1 or anti mouse OX40L monoclonal antibodies and acquired by flow cytometry, cells were first gated on their FSC/SSC profile before PD-L1 or OX40L expression was analysed. For PD-L1 silencing values are expressed as MFI percentage of control which was normalised to 100%. In each case mean and SEM is shown (n=3 experimental repeats). (**C,D**) C57/Bl6 mice were subcutaneously implanted with B16F10 tumour cells ($1x10^6$/per tumour) (n=8). Tumours were allowed to develop until palpable (day 5) at which point each tumour was injected with either Buffer, siNeg/pNeg, siPDL1 (siPDL1), pOX40L (pOX40L) or siPDL1/pOX40L. Tumour growth was monitored and subsequent doses were administered at days 7 and 11 in accordance with (**C**). The tumour growth rate is shown in (**D**). Mean and SEM is shown in each case, statistical analysis was carried out using students T test *$p<0.05$, **$p<0.005$, ns non-significant.

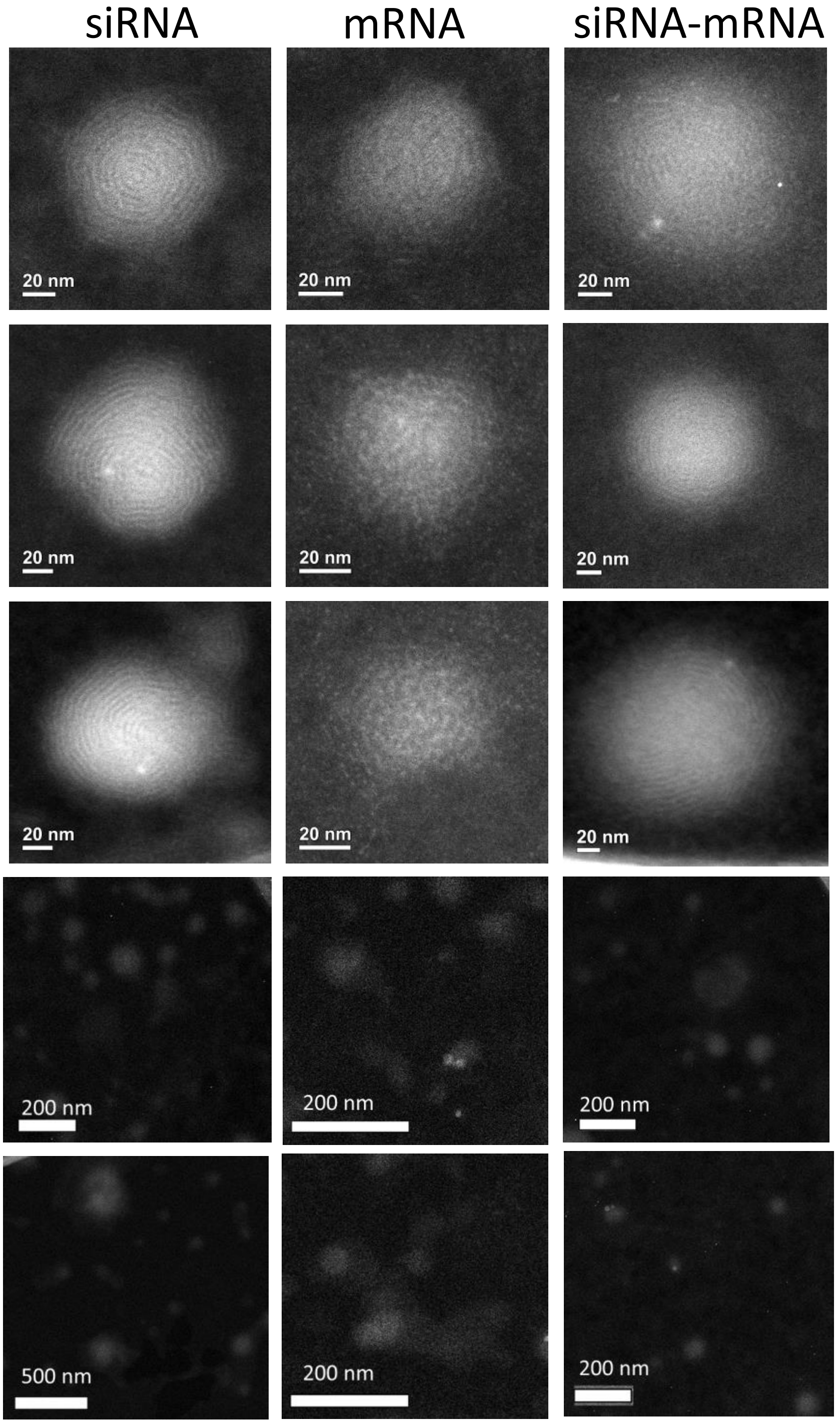

Supplementary Figure 2. Annular Dark Field Scanning Transmission Electron Microscopy (ADF-STEM) images of SNALPs. SNALPs were formulated with siRNA, mRNA or a combination of both RNA molecules as previously described. SNALPs were dropcasted on to a graphene grid and imaging was performed using a Tecnai Osiris transmission electron microscope. Images represent either a single event or a region of interest representative of the wider field. SNALPs have shown an irregular structure with evidence of internal concentric rings.

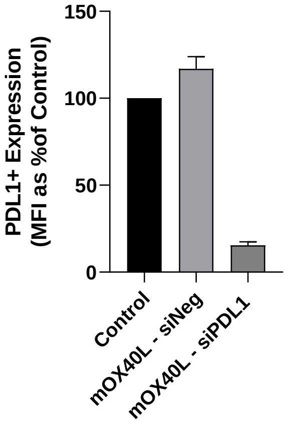

Supplementary Figure 3. PDL1 knockdown is a specific effect of siPDL1 transfection. To confirm PDL1 down regulation is specific rather than an artefact of transfection, B16F10 cells were transfected with SNALPs containing mOX40L and non-specific siNeg or mOX40L and siPDL1 (0.75 μg). After 48h cells were harvested and double stained with fluorescently labelled anti mouse OX40L and PDL1 monoclonal antibodies. The values obtained for PDL1 silencing, expressed as MFI percentage of control normalized to 100%, is shown.

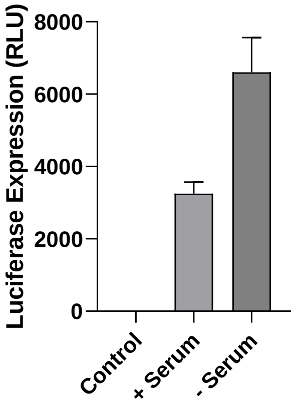

Supplementary Figure 4. SNALP transfection occurs in the presence of serum, though is less efficient. B16F10 cells were transfected with SNALPs containing mLuc (0.75 μg) in the presence or absence of serum containing media (RPMI + 10% FCS) as previously described. A condition was left transfected as a control. After 48h cells were harvested and lysed with reporter lysis buffer. Luciferin was added to cell lysate and the luminescence was detected with plate reader (BMG LABTECH, FLUOstar® Omega).

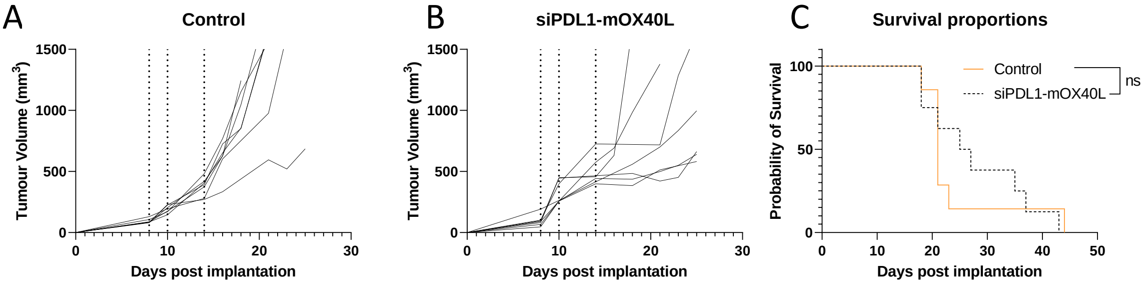

Supplementary Figure 5. Combinatory SNALPs does not delay tumour growth or improve overall survival in difficult to transfect CT26 model. CT26 cells ($1x10^6$/per mouse) were implanted subcutaneously into the lower lateral flank of BALB/c mice (n=7-8 per group). Once the tumours reached c.5 mm in diameter (day 8) and at days 10 and 14 post implantation mice were injected i.t. with SNALPs containing both mOX40L and siPDL1 (13 µg, siPDL1-mOX40L), or injected with buffer (Control). The tumour growth was recorded until the humane end points were met. For each group the data is presented as a spaghetti plot for individual mice (**A-B**). Mouse survival over the course of the experiment is plotted as a Kaplan-Meier plot (**C**). Tumour size data is presented as mean ± SEM. Survival data was analysed using a Mantel-Cox test, ns nonsignificant.
